# Global predictions of primary soil salinization under changing climate in the 21st century

**DOI:** 10.1038/s41467-021-26907-3

**Published:** 2021-11-18

**Authors:** Amirhossein Hassani, Adisa Azapagic, Nima Shokri

**Affiliations:** 1 Department of Chemical Engineering and Analytical Science, The University of Manchester, Sackville Street, Manchester, M13 9PL UK; 2 NILU - Norwegian Institute for Air Research, PO Box 100, Kjeller, 2027 Norway; 3 Institute of Geo-Hydroinformatics, Hamburg University of Technology, Am Schwarzenberg-Campus 3 (E), 21073 Hamburg, Germany

**Keywords:** Climate change, Biogeochemistry, Environmental chemistry, Hydrology, Hydrology

## Abstract

Soil salinization has become one of the major environmental and socioeconomic issues globally and this is expected to be exacerbated further with projected climatic change. Determining how climate change influences the dynamics of naturally-occurring soil salinization has scarcely been addressed due to highly complex processes influencing salinization. This paper sets out to address this long-standing challenge by developing data-driven models capable of predicting primary (naturally-occurring) soil salinity and its variations in the world’s drylands up to the year 2100 under changing climate. Analysis of the future predictions made here identifies the dryland areas of South America, southern and western Australia, Mexico, southwest United States, and South Africa as the salinization hotspots. Conversely, we project a decrease in the soil salinity of the drylands in the northwest United States, the Horn of Africa, Eastern Europe, Turkmenistan, and west Kazakhstan in response to climate change over the same period.

## Abstract (web summary)

Excess salt accumulation in the root zone causes soil salinization influencing soil health, biodiversity and food security. Authors used machine learning algorithms to predict global scale soil salinization under changing climate in the 21st century.

## Introduction

The Soil Science Society of America^1^ defines saline soil as a non-sodic soil containing sufficient amount of soluble salt which could adversely influence most crop plants. Conventionally, electrical conductivity of a saturated soil paste extract (EC_e_) has been used as a measure of the soil salinity^2^. Soil salinization is a land degradation process that results in excessive accumulation of soluble salts in the soil^3,4^. In naturally occurring or primary soil salinization, the predominant origins of soluble salts are rainfall (wet deposition of oceanic salts), aeolian processes (dry deposition of oceanic salts), and physical or chemical weathering of parent rock materials^5,6^. Transport of the accumulated salts from saline geological depositions by streamflow or shallow underground waters is an additional source of primary salinization^7^. In anthropogenic or secondary soil salinization, however, the main sources of salinization are human interventions, such as irrigation with brackish or saline water, rising water tables due to poor land and water management, surface or subsurface sea water intrusion into coastal aquifers as a result of rising sea levels or over-exploitation of the fresh underground waters, and overuse of fertilizers^5,7,8^.

Excessive accumulation of the soluble salts in the root zone may go beyond the salt tolerance of plants, affecting adversely the growth rate of the plants^9^. A soil with salinity of EC_e_ ≥ 2 dS m^−1^ (at 25 °C) is traditionally considered as a saline soil^10^; however, depending on the plant type, climatic conditions, and soil-water balance properties, the salt tolerance of sensitive crops and plants can be different^11^. Salinity stress deteriorates the plants’ transpiring leaves which is known as specific ion effects^12^ or directly reduces the plant water uptake from the rooting zone, resulting in osmotic stress on the plant^13,14^. Soil salinity also imposes nutritious imbalances in plants^6^. Soil salinity between 2 and 4 dS m^−1^ can negatively impact the yields of sensitive plants and at salinity levels higher than 8 dS m^−1^, the growth of most of crops and plants shows a severe decrease in response to excessive soil salinity^1,15^. Vegetation loss in turn reduces the soil stability and exposes the soil to wind or water erosion^16^. In addition to deleterious effects on vegetation, excessive soil salinity decreases the biological functioning of the soil micro-organisms to a level that disturbs the soil nitrogen cycle, respiration, and organic matter input^17,18^. Reduced environmental health due to aeolian dispersion of saline dust originated from the saline soils^16,19^, land abandonment and desertification^20,21^, worsening of economic welfare, and human migration are other detrimental consequences of excessive soil salinity^6,19^.

Accurate and reliable data on spatial distribution of salt-affected soils are important to develop action plans for management of soil, water, and vegetation and will contribute toward data-driven policy making^22–24^. These data have also implications for tuning large-scale agro-ecological models^25^ and planning sustainable reclamation practices^26^. With varying levels of accuracy and spatial coverage, from the local^27–29^ to the global scale^30–33^, defining the spatial distribution and location of salt-affected soils has been under focus of various studies. According to the global-scale studies, salt-affected soils lie across all climate zones and continents with an estimated global area of ~8.31–11.73 Mkm^2^, depending on the methods used for estimation of area of the salt-affected soils. Nevertheless, the general consensus is that the saline and salt-affected soils (including sodic soils) are particularly found in drylands where the excess of evaporation over water input to the soil accumulates salts in the upper soil layer^3,34,35^.

Drylands, including hyper-arid, arid, semi-arid, and dry sub-humid lands, are characterized by a multi-annual Aridity Index (AI) of less than 0.65 mm mm^−1^, computed as the ratio of total precipitation to potential evapotranspiration^36,37^. Drylands occupy a total of ~45% of the Earth’s surface^38,39^. With the advance of proximal/remote sensors and digital soil mapping techniques, there is a rising interest in spatio-temporal mapping and monitoring of the soil salinity^40–42^. Due to the temporal and vertical variability in salinity levels of the salt-affected soils^5,42^, updated predictions on long-term variations of soil salinity can provide a clearer understanding of the dynamics of the terrestrial carbon sink^43^, climate change impacts^44^, and alterations in the land, vegetation, and water resources^45^. Even though the above-mentioned purely spatial or spatio-temporal studies have substantially advanced our understanding of the current status of the salt-affected soils and processes involved in salinization, predictions of the future extent and dynamics of soil salinization at the global scale are still missing, partly due to the complex processes and many parameters influencing soil salinization at the global scale. This makes the future prediction of soil salinization in the face of future climate uncertainties a grand challenge, which is precisely one of the key objectives of the present investigation.

The projected hydrological consequences of climate change may result in physical, biological, biochemical, and chemical degradation of the soils^46^. As one of the major threats to soil stability, fertility, and biodiversity, it is expected that the soil salinity will be a significant and growing concern in a warmer world^47,48^. To formulate appropriate plans for sustainable management of soil, water, and vegetation, reliable predictions on the probable occurrence and expansion or shrinkage of the salt-affected soils in response to the threat of climate change are crucial. Compared to other dynamic soil properties, such as P, N, and organic matter content, prediction of soil-salinity responses to climate variability on a global scale has received much less attention^49^. The available literature on the effect of climate change as a source of soil salinization is mainly descriptive and quantitative predictions of the future status of salt-affected soils on the basis of current trends are rare. The IPCC report^50^ predicts that climate change will likely impact all the primary mechanisms for soil salinization, including soluble salts accumulation due to a change in hydrological balance, sea salt intrusion, and wind-born salt deposition. An increase in the rate of evapotranspiration and alteration in precipitation patterns, particularly in arid and semi-arid areas, is expected to reduce the soil leaching efficiency and consequently, increase the salt concentrations in top-soil horizons^51–53^. Expansion of irrigated areas and the higher demand for water use under rising global temperatures, in combination with poor drainage/irrigation practices, are expected to result in the spread of secondary salinization^54^. Land use modifications and occurrence of more extreme climate events, such as prolonged droughts followed by severe floods, have the potential to release and redistribute large amount of salts from the geological substrates with high concentration of salts and may put new areas at risk of soil salinization^55^. In addition, rising sea levels and unsustainable extraction of freshwater resources from coastal aquifers can worsen the issue of sea water-induced soil salinization in coastal regions^53,56^.

A few studies investigated some aspects of the relationship between projected climate change and soil salinization. Szabolcs^51^ was among the first who estimated that the salt-affected areas in North Mediterranean regions will be doubled by 2050 in response to 1 °C increase in the average annual temperature. Similarly, National Land and Water Resources Audit^45^ estimated that Australia’s drylands at risk of soil salinity imposed by dryland management actions may expand to 170,000 km^2^ in 2050, relative to approximately 57,000 km^2^ in 2000. Schofield et al.^57^ developed a set of soil salinization indicators including low relief, high two-way annual moisture flux, and local flow deficit in large catchments to identify the current and future (2079–2099) locations with salinization potential across the globe and concluded that areas at risk of soil salinity are expanding. Although these studies provide an understanding of the salinization potential and limitations of the methods used for projecting the soil salinity, they are not based on up-to-date datasets and they mainly highlight the areas at risk; no quantitative and spatially explicit predictions are provided. Other studies on predicting impacts of climate change on soil salinization are mainly focused on predicting secondary salinization processes imposed by unsustainable irrigation practices^58–60^ or sea water intrusion^61–63^ at local scales. Thus, there is a need for a quantitative global-scale analysis, characterizing the geographical distribution and projecting the long-term variations in soil salinity in the face of future climate fluctuations and uncertainties, which motivated the present investigation.

This study is among the initial attempts for addressing the need for a quantitative tool capable of predicting long-term primary soil salinity on a global scale with a high spatial and temporal resolution. These models and the results will be of interest to local authorities, land managers, and policy makers, helping to plan mitigation of and adaptation to soil salinization. In particular, we performed comprehensive data-driven modelling and analyses to reveal how the projected or hypothesized variations in the key drivers may influence primary soil salinity on the global scale, in both mid- (2031–2060) and long-term (2071–2100) futures. We only focus on soil salinity in the top-soil horizon (0–1 m), quantified by the concentration of soluble salts which is expressed by the extent of EC_e_. Other aspects of salt-affected soils, such as sodicity (which is traditionally measured by the soil exchangeable sodium percentage) or alkalinity, are not within the scope of this analysis. The potential soil salinity caused by sea level rise, saline groundwater, or irrigation is also excluded from the study. Note that modelling the salinity intrusion in coastal areas in response to sea rise needs a relatively precise estimation of the future groundwater extraction from the coastal aquifer. Similarly, projected data of groundwater level and salinity change (either natural or anthropogenic) are needed for predicting the groundwater-induced soil salinity, which is not currently available. As mentioned in Yeo^54^, it is difficult to generate a clear prediction of the impacts of climate change on the extent of salinization caused by irrigation as this requires reliable estimations of irrigation expansion and the quality of irrigation water in future. Therefore, this study can be deemed as projection of the primary soil salinization under future climate uncertainty.

Several numerical methods have been developed to simulate the soil salinization by considering different modes of mass transfer mechanisms transporting solute in unsaturated soil (such as Corwin et al.^64^, Schoups et al.^65^); however, the application of these models remains limited to small-scale simulations where the detailed soil characteristics data are available. Moreover, employing analytical approaches, such as the stochastic model of soil salinity^66,67^ or the developed frameworks for mechanistic modelling of the climate, vegetation, and soil salinity interactions^68–70^, would be applicable for projecting soil salinity only if the initial soil salinity or required calibration parameters for tuning were available; currently, such data are not available, particularly on a global scale. As a result of these practical limitations, we utilize Machine Learning (ML) algorithms as an alternative approach to predict the future of primary soil salinization on a global scale.

Recent studies demonstrated the great potential of ML algorithms in digital soil mapping and predicting spatio-temporal properties of the soil^71^. In the present study, we used supervised ML algorithms for projecting the long-term (up to year 2100) variations in soil salinity. In summary, the methodology included exposure of a known set of input data (predictors) and a set of known responses (soil salinity profiles) to ML models to develop trained models based on the relations between the two sets. The trained models were later applied to a new set of known input data (with unknown responses) to generate predictions for the response (see Methods).

Dryland areas are generally known as the regions with the highest vulnerability to hydro-climatic consequences of climate change^7^. For this reason, the majority of our measured input soil-profiles data were sampled from the dryland areas of the world. We made predictions of soil EC_e_ only for the dryland areas with an AI ≤ 0.65^37^ as extrapolation of the ML results to other areas is a matter of uncertainty^72^. The rest of this paper discusses the significance of the predictors and global variation in primary soil salinization at the grid-cell level, followed by the country-level analysis. Changes in the total area of drylands with an EC_e_ ≥ 2 dS m^−1^ (and EC_e_ ≥ 4 dS m^−1^) at the country and continental levels are also presented. Finally, methods and their limitations are discussed.

## Results

### Predictors’ significance and their relation to the predicted soil salinity

Supplementary Table 1 shows the estimates of the predictor importance for the trained models based on the output of the GCMs used for spatio-temporal prediction of the EC_e_ (see Methods for details of predictors and trained models). The percentage values reported in Supplementary Table 1 indicate the relative importance of each predictor in the final trained model in each input dataset. Among the 14 applied predictors, the long-term (5-year average) annual precipitation frequency is relatively the most influential soil predictors with an overall importance of 14% for all 16 best-fitted models. WRB soil classes and daily evapotranspiration are, respectively, the second and the third influential environmental predictors in estimation of the soil EC_e_ with the overall importance of 13.07% and 9.26%, respectively.

The effect of each of the 13 non-categorical predictors (see Methods) on the predicted outcome of the trained models is shown in Supplementary Fig. 1 (Partial Dependency Plots, PDPs). The effect of long-term daily wet and dry deposition rates of sea salts are presented in Supplementary Fig. 2. Supplementary Fig. 1a, b suggest that shallower depths are not necessarly associated with higher EC_e_ in soil under natural conditions. However, in many previous experimental, analytical and numerical investigations^73–77^, higher solute concentrations and solute precipitation close to the evaporation surface were observed when the Peclet number (quantifying the relative importance of chemical diffusion and advection) was greater than the one during saline water evaporation from porous media. It must be noted that under natural environmental conditions (which is the case in our investigation), many parameters influence the complex dynamics of solute transport and deposition in soil, including the vegetation and land cover, rainfall, micro-organisms’ activities, depth of water tables, soil chemical compositions and heterogeneity, human interventions, and land-atmosphere interactions. These parameters, which could not be included in the majority of the previous experiments conducted under well-controlled laboratory conditions or numerical simulations, could induce significant impacts on solute distribution in soil under natural conditions^3,29^.

Fine-textured soils (soils with the higher clay content) show higher Water Holding Capacity (WHC, the difference between field capacity and wilting point) and lower saturated hydraulic conductivity. Overall, the predicted EC_e_ values provided by each of the 16 trained models show a reverse relation with the soil clay content and WHC which is in line with previous experimental results^78^ and a literature review^78^ (Supplementary Fig. 1c, g, h). Similarly, based on numerical, experimental, and field-scale investigations, Shokri‐Kuehni et al.^79^ concluded that soil salinity for coarse‐textured soils is greater than for medium and fine‐textured soils when the water table is shallow and hydraulically connected to the evaporation surface. Our predicted results regarding the effects of soil texture on soil salinity are generally in agreement with the above-mentioned physically based determined trends and behaviour.

Moreover, the analysis of PDPs shows that the effective plant rooting depth influences the predicted EC_e_ approximately up to the depth of 4 m. The PDPs also demonstrate a strong negative correlation between soil salinity and terrain elevation, topographic slope, and precipitation frequency (Supplementary Fig. 1d, e, i). These correlations can be explained by the prior pedologic knowledge: the lower hillslope and the higher precipitation frequency result in more efficient leaching of the salts accumulated in the root zone^66^, resulting in lower salinity. The relationship between the predicted soil EC_e_ values and other predictors, however, is more complicated and deriving general trends remains a challenge.

### Projected soil salinity in drylands up to the year 2100

**Fig. 1:**
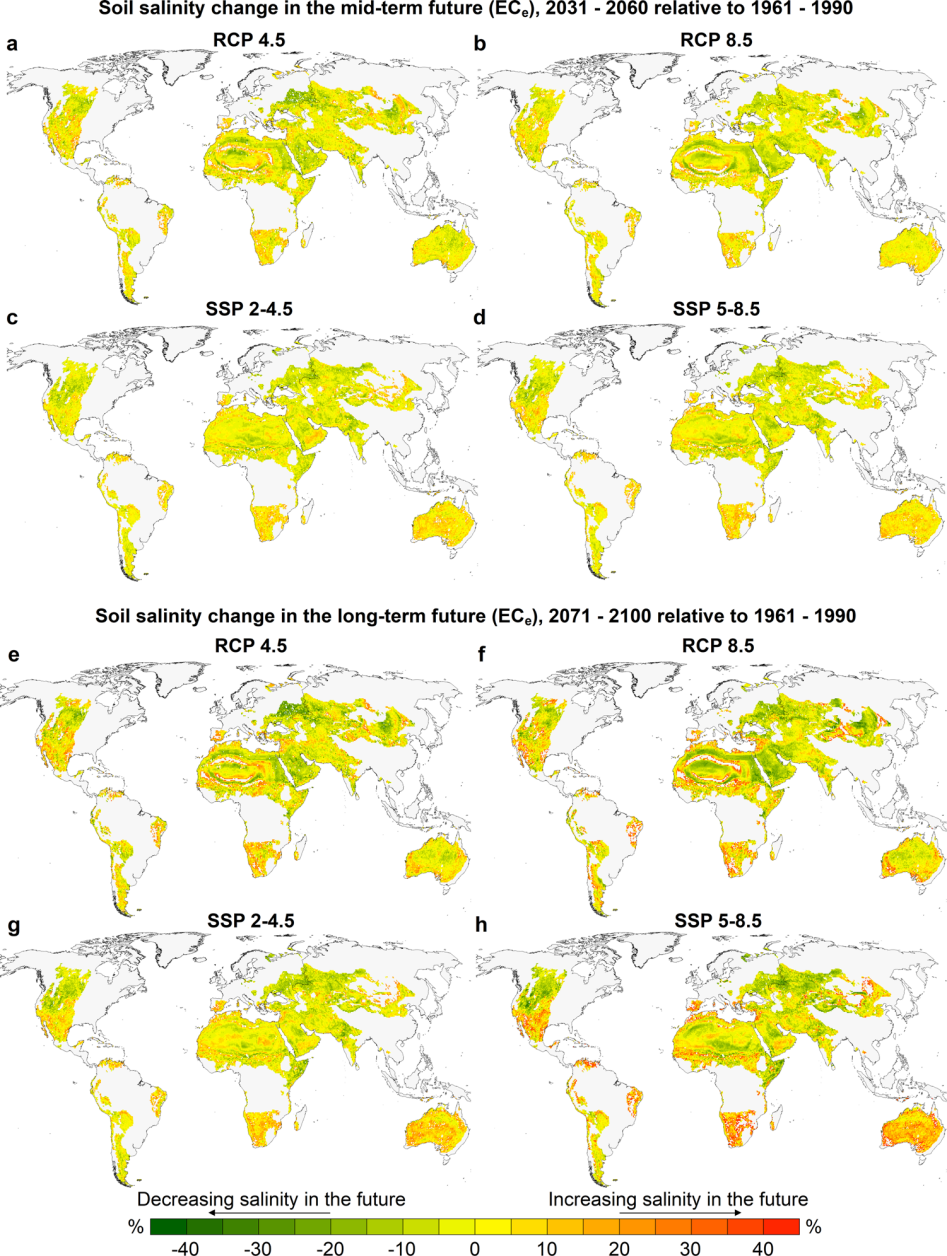
Multi-model ensemble mean of the change in predicted soil salinity represented by saturated paste electrical conductivity (EC_e_) in the mid- and long-term futures, relative to the reference period (1961–1990) under different greenhouse gas concentration trajectories. **a**–**d** Mid-term prediction of changes in EC_e_ (2031–2060). **e**–**h** Long-term prediction of changes in EC_e_ (2071–2100). The average of the predictions to the depth of 1 m were used for calculations of salinity change. At each map cell (pixel) and based on each GCM, we calculated the mean of soil salinity for the reference, mid-, and long-term future periods and then computed the relative change as: (Future mean − Reference mean)/Reference mean; the percentage value of each cell represents the multi-GCM mean of the calculated relative changes presented by the colour map. Positive values indicate an increase in soil salinity while the negative values are indicative of a decreasing trend.

The trained models based on the output of Global Circulation Models (GCMs) were applied to new input predictor data to estimate the annual soil salinity for each grid-cell (0.5° spatial resolution) of the global soil base map of the drylands between 1904 and 2100 (see Methods for details of GCMs, predictors, and trained models). Figure 1 shows the spatial distribution of the change in primary soil EC_e_ projected by the multi-model ensembles in the mid- (2031–2060) and long-terms (2071–2100), relative to the reference period (1961–1990) at the 0.5° spatial resolution. The RCP 4.5 and RCP 8.5 scenarios (Representative Concentration Pathways which result in a respective radiative forcing of 4.5 and 8.5 W m^−2^ in year 2100, relative to pre-industrial conditions) are related to CMIP5 (Coupled Model Inter-comparison Project Phase 5^80^) data project, while the SSP 2-4.5 and SSP 5-8.5 scenarios (projections forced by RCP 4.5 and RCP 8.5 global forcing pathways for the Shared Socio-economic Pathways 2 and 5) refer to CMIP6 (CMIP Phase 6^81^).

Our results reveal that the sign (positive: indicative of a higher EC_e_ and negative: indicative of a lower EC_e_) and intensity of changes in primary soil salinity are geographically highly variable; the variations are more extreme at the end of the 21th century compared to the mid-term future. Generally, the relative changes in soil salinity are more severe for the GHG emission rates which result in higher radiative forcing scenarios (RCP 8.5 and SSP 5-8.5). However, the intensity and spatial distribution of the projected changes based on the CMIP5 models are not necessarily the same as the CMIP6-based models predictions. Although our aim was to include all available projections in the analysis, in the case of discrepancy between CMIP5 and CMIP6 models, the predictions made based on the CMIP6 GCMs should be prioritized as they are more recent, forced by more updated data, and generally of higher spatial resolutions^81^.

According to our long-term predictions based on all multi-model ensembles, the drylands areas of South America, southern Australia, Mexico, south-west United States, and South Africa are generally at the highest risk of increased soil salinity, compared to the reference period. The threat of climate-induced soil salinity is also projected to increase in drylands of Spain, Morocco, and northern Algeria. To a lesser extent, western and southern Sahara and central Indian drylands, in addition to the desert soils of southeast Mongolia and north of China, are estimated to become saltier in response to the projected climate change by 2100 for different GHG concentration trajectories. On the other hand, our results indicate that the extent of soil salinity will remain constant or decrease relative to the reference period in the drylands located across the northwest United States, the Horn of Africa, Eastern Europe, Turkmenistan, and west Kazakhstan.

Additionally, Supplementary Fig. 3 shows the long-term future relative change in the five-year moving averages of daily dry and wet deposition rates of the sea salts (the 1971–2100 mean minus the 1961–1990 mean) projected by the multi-GCM ensemble means, as the two predictors used for training the models. Overall, the CMIP6 models predict a more severe increase or decrease in dry and wet deposition rates; however, all ensemble means are in agreement on an increasing trend in the dry deposition rate of sea salts in coastal regions, particularly in the southern hemisphere. All models also project a decreasing trend in dry deposition rates in north-western United States, west Canada, and central Asian regions; however, for these locations, the projected sign of the change in wet deposition rates is different between the CMIP5 and CMIP6 models. To some extent, the projections of these deposition rate can explain why soil salinity decreases in some regions, e.g. central Asia and Kazakhstan, where there is less certainty on the projected sign of changes in precipitation and evapotranspiration^82^.

**Fig. 2:**
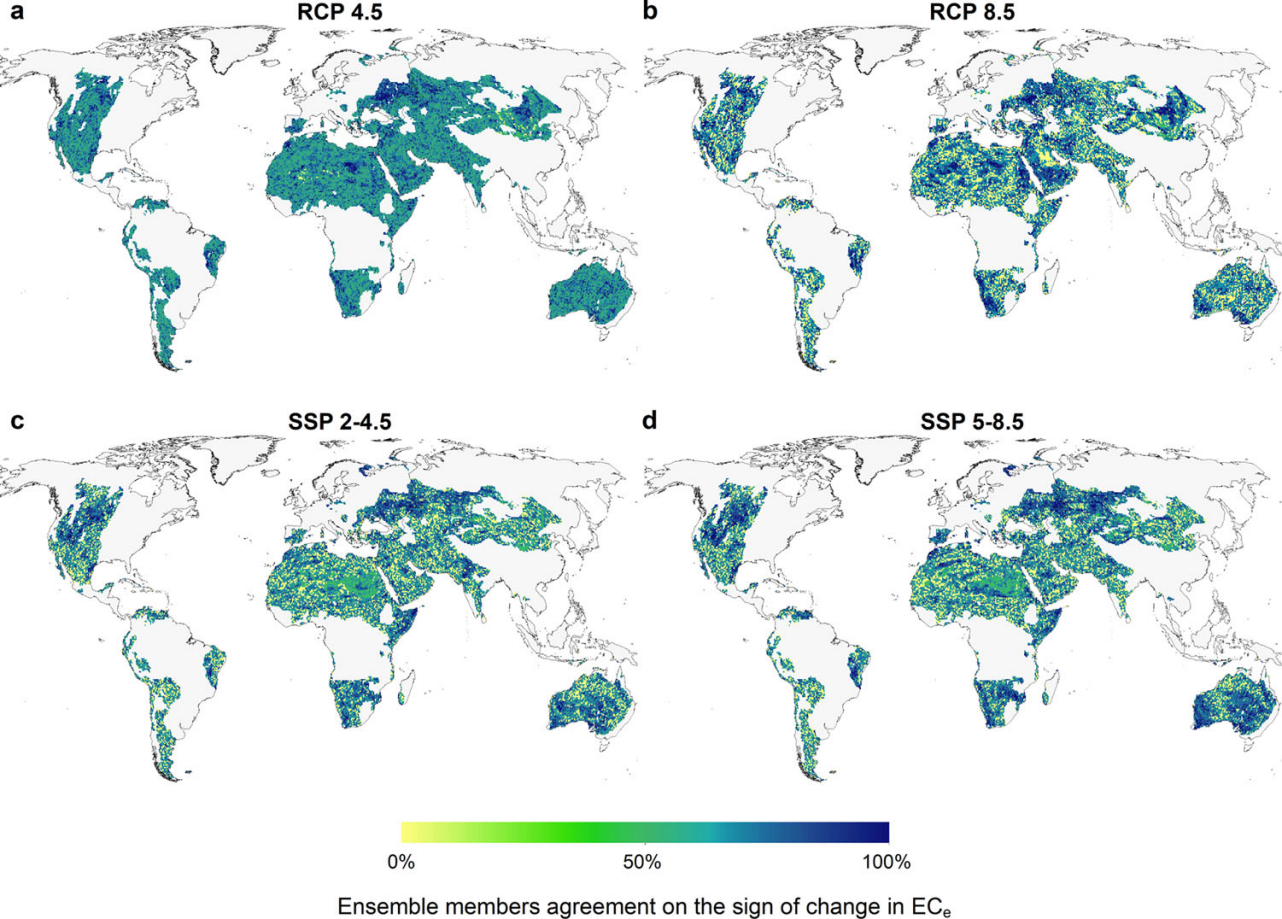
Multi-GCM ensemble agreement on the sign of change in predicted values of soil EC_e_ in the long-term future (2071–2100), relative to the reference period (1961–1990) under different greenhouse gas concentration trajectories. **a**, **b** Multi-GCM ensemble agreement of the models adopted from Coupled Model Inter-comparison Project Phase 5 (CMIP5) forced by RCP 4.5 and RCP 8.5 scenarios (Representative Concentration Pathways, which result in a respective radiative forcing of 4.5 and 8.5 W m^−2^ in year 2100, relative to pre-industrial conditions), respectively. **c**, **d** respective multi-GCM ensemble agreement of the models adopted from CMIP6 project under SSP 2-4.5 and SSP 5-8.5 pathways (projections forced by RCP 4.5 and RCP 8.5 global forcing pathways for the Shared Socio-economic Pathways 2 and 5, respectively). 100% shows the full agreement of the models on the sign of change, while zero indicates inconsistency among the models’ predictions.

Not all of the predictions generated based on the CMIP5 and CMIP6 GCMs used in this study are in agreement on the extent and sign of the soil salinity by the end of the century. Figure 2, in particular, shows the multi-model ensemble agreements on the sign of the predicted change in soil salinity in the long-term future under different trajectory scenarios of GHG concentration. A cell value close to 100% indicates a complete agreement of the ensemble members on the sign of the salinity change. For the RCP 4.5 ensemble, as an example, an ensemble agreement of 100% of a grid-cell shows that all seven models in the ensemble are predicting an increase or a decrease in soil salinity in the long-term future relative to the reference period (depending on the sign of change). Especially under the SSP 2-4.5 and SSP 5-8.5 scenarios, the multi-GCM certainty of the predictions for a great proportion of the drylands of southern/eastern Australia, South America, and southern Africa indicate the southern hemisphere is at a higher risk of salinity caused by climate change. The projected increase in soil salinity in south-west and southern Australia induced by rising shallow groundwater tables as a result of dryland resource management and activities^45^ can exacerbate the climate-induced soil salinization projected here. However, the certainty of the predictions made for drylands located in the Middle East, Russia, and Sahara is seemingly lower than for the other zones. For those dryland regions, the uncertainty is also recognizable through the difference in the predictions made based on the CMIP5 and CMIP6 models in Fig. 1. For example, the CMIP5 models predict an increase in soil salinity in Russian drylands, while the CMIP6 models show the opposite trend in those regions.

### Country-level projected changes in soil salinity

At the country level, we calculated descriptive statistics for the relative changes in soil salinity estimated at each grid-cell (in the mid- and long-term futures compared to the reference period) based on the multi-model ensemble mean, including grid-cells mean, 95% confidence intervals of the mean, standard error of the mean, and variance (Supplementary Tables 2–9). We did not calculate these descriptive statistics at the continental level as there was no noticeable difference between the results for various continents due to the high number of grid-cells within each continent.

Although the country-level results mask the majority of the local-scale variabilities of the soil salinity, the provided statistics help to have a better understanding of the countries with the highest risk of salinization. We ranked the countries based on the total number of grid-cells located in each country and calculated all aforementioned statistics only for the 30 countries with the highest number of grid-cells (Supplementary Table 10 shows the top 30 countries and the total estimated area of their drylands).

For the 2071–2100 period relative to 1961–1990 and under RCP 8.5 as the worst case scenario, the countries with the highest relative increase in the soil salinity were Brazil (with a mean grid-cell increase in EC_e_ of 15.1% and the 95% confidence intervals of 13.25–16.95%), Namibia (13.57%; 12.1–15.04%), South Africa (11.2%; 9.41–13%), and Mexico (6.38%; 4.96–7.8%). The increase in soil salinity for Australia was much lower (3.31% and 2.88–3.73%). Under SSP 5-8.5, the countries with the highest relative increase in grid-cell means of soil salinity in the same period were Botswana (24.94%; 22.71–27.16%), South Africa (21.35%; 19.84–22.85%), Namibia (17.69%; 16.14–19.24%), and Brazil (16.21%; 14.77–17.66%). Overall, our calculated statistics suggest that the soil salinity will be increased more extensively by the climate change impacts in the regions spread across the southern latitudes, specifically below −20°.

### Change in the total area of salt-affected soils in drylands

**Fig. 3:**
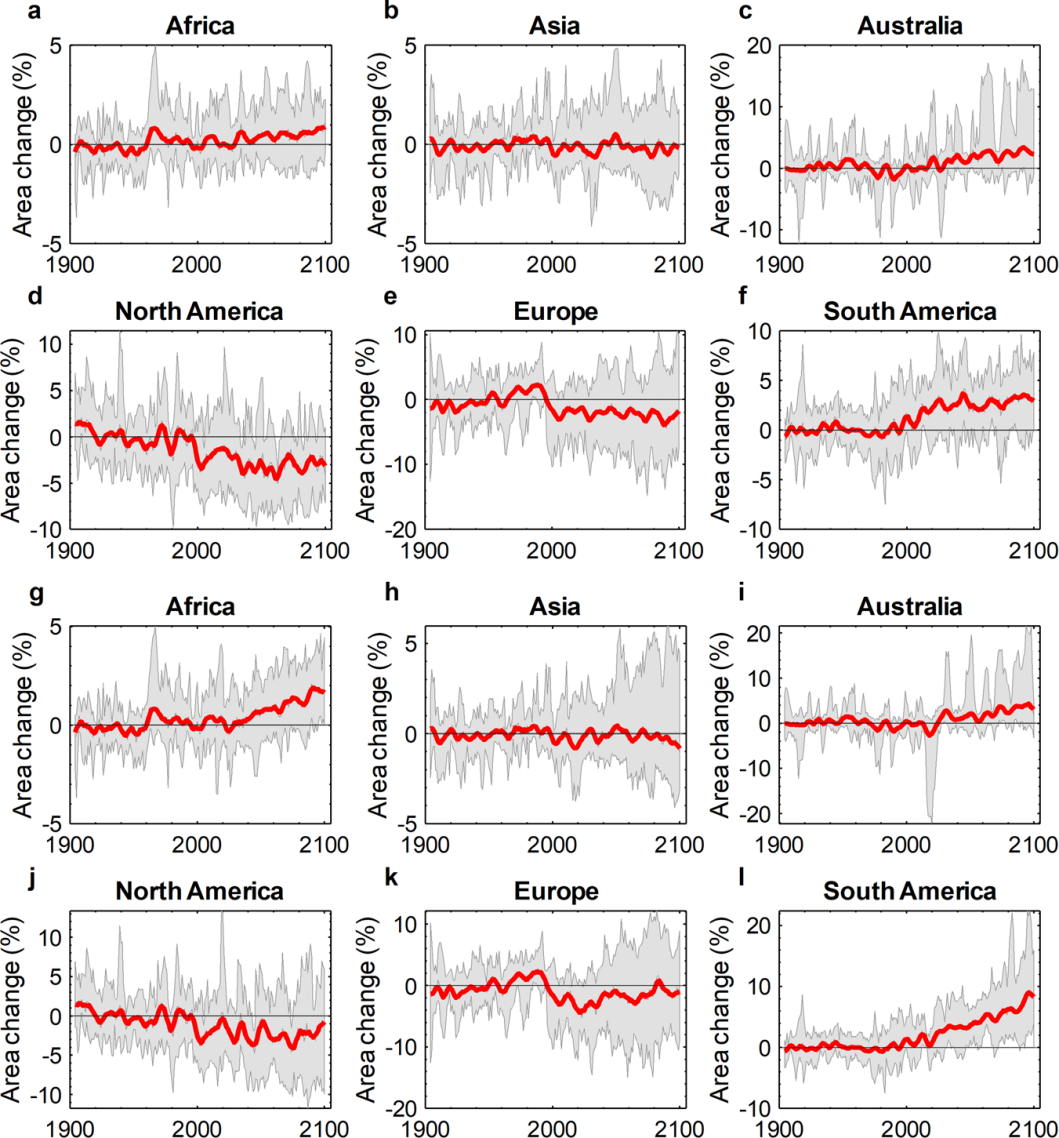
Continental-level predicted annual change in the total area of soils with an EC_e_ ≥ 2 dS m^−1^ relative to the 20th century average (1904–1999) for the models obtained from the CMIP6 data project. **a**–**f** Relative change under SSP 2-4.5 greenhouse gas concentration trajectory. **g**–**l** Relative change under SSP 5-8.5 greenhouse gas concentration trajectory. Shaded areas show the minimum and maximum range of the relative changes predicted by multi-model ensemble members. Red lines show the low-pass filtered (5-year running window) of the multi-model ensemble mean of the predicted variations; since all spatio-temporal predictors are five-year moving averages, 1904 is the beginning of the period.

Additionally, based on our predictions for soil salinity extent in each grid-cell, we estimated the total area of salt-affected soils up to year 2100. Currently, no unique definition is available for the salt-affected soils. Contingent on the soil classification system, different values of EC_e_, ranging from 2 dS m^−1^ to even 30 dS m^−1^, are adopted as the minimum threshold of salinity for characterizing the saline soils^35,83,84^. Accordingly, here we quantified the areal variation of the soils exposed to the threat of primary salinization assuming an EC_e_ equal to 2 dS m^−1^ as the critical threshold, corresponding to the upper salinity limit tolerable by sensitive crops^11^. The results were computed at the country (Supplementary Table 11), continental (Table 1, Fig. 3; Supplementary Fig. 4), and global levels (Supplementary Fig. 5). Additionally, Supplementary Figs. 6–8 and Supplementary Tables 12, 13 show the projected variation in the total area of naturally occurring salt-affected soils assuming 4 dS m^−1^ as the critical threshold at the continent and country levels. As before, at the country level, only the top 30 countries with the highest number of the grid-cells were included. This analysis could be an indicator of the spatial expansion of the soil salinity in drylands in response to climate change.

**Table 1:** Continental-level predicted change in the total area of soils with EC_e_ ≥ 2 dS m^−1^ in the mid- and long-term futures relative to the average of the 1904–1999 period under different greenhouse gas concentration trajectories.

Scenarios	Africa	Asia	Australia	North America	Europe	South America
RCP 4.5, mid term (%)	0.00	−1.03	0.02	−0.23	−6.58	2.35
RCP 4.5, long term (%)	0.17	−2.02	0.70	−0.33	−9.13	1.84
RCP 8.5, mid term (%)	0.02	−1.36	0.79	0.13	−2.55	2.21
RCP 8.5, long term (%)	−0.02	−3.05	0.60	0.83	−5.35	4.88
SSP 2-4.5, mid term (%)	0.41	−0.05	1.59	−3.32	−2.09	2.56
SSP 2-4.5, long term (%)	0.61	−0.25	2.40	−2.89	−2.68	3.04
SSP 5-8.5, mid term (%)	0.51	0.02	1.36	−2.28	−1.90	3.60
SSP 5-8.5, long term (%)	1.45	−0.28	3.38	−2.45	−0.92	6.70

Overall, under emission rates resulting in the radiative forcing of 8.5 W m^−2^, all CMIP5 and CMIP6-derived predictions indicate an increasing trend in the total area of dryland soils with an EC_e_ ≥ 2 dS m^−1^ for Australia and South America and a decreasing trend for Asia and Europe relative to the average of 1904–1999 period. For Australia and South America, we estimate the respective increases of 3.4% and 6.7% in the total area of dryland soils with EC_e_ ≥ 2 dS m^−1^ between 2071–2100 relative to 1904–1999 period according to the multi-GCM ensemble means under the SSP 5-8.5 scenario. The CMIP5 and CMIP6-derived predictions of the total area of dryland soils with EC_e_ ≥ 2 dS m^−1^, however, are not in agreement on the sign and extent of the change for Africa and North America. The multi-model ensemble means under the SSP 5-8.5 scenario predict an increase of 1.5% and a decrease of 2.5% for the total area of dryland soils with a salinity ≥2 dS m^−1^ located in Africa and North America (between 2071–2100 relative to 1904–1999), respectively. Brazil (with 43%), Mexico (14.5%), and Mongolia (8%) had the highest estimated expansion in the total area of dryland soils with a salinity ≥2 dS m^−1^ between 2071–2100 relative to 1904–1999 periods under SSP 5-8.5 at the country level. On the opposite side of the continuum, Canada (with −10%), Somalia (−8.5%), and Ethiopia (−5%) had the largest predicted shrinkage of saline soils under SSP 5-8.5 (among the top 30 counties with the highest number of grid-cells in our analysis).

## Discussion

One of the questions that arises from this research is if the projected changes in primary soil salinization can actually occur in the time scales (10–40 years or 50–80 years) used for projections, especially in inland (hyper) arid regions where salt deposition is minimal and weathering very slow. The fast transition in near surface salt-budget has been reported in some studies which evaluated the temporal variations of naturally occurring soil salinity in arid environments using laboratory analysis and remote sensing techniques. Bannari and Al-Ali^85^ examined the effect of climate change on spatio-temporal variability of soil salinity during the last 30 years (1987–2017) in the state of Kuwait using Landsat images and 100-geo-referenced soil data; for instance, only between 1987 and 1992, they estimated an increase equivalent to 350% in total area of salt-affected soils compared to the salt-affected area approximated in map of 1987 (433 km^2^). As another example, Wang et al.^86^ investigated the spatio-temporal changes of soil salinity in Kashgar region, north-western China with respective annual precipitation and potential evapotranspiration of 67.5 mm and 2100 mm using multi-temporal Landsat images and saline soil types from 19 field survey sites in the years 2000, 2010, and 2017. They estimated a total of 6.13% decrease (relative to 26,500 km^2^) in the net area of salt-affected soils between 2000 and 2010, followed by further decrease of 1.75% between 2010 and 2017. Another example is the study by Taghadosi and Hasanlou^87^ who monitored the salinity changes in bare soils near the arid district of Bakhtegan Lake in Iran between 2000 and 2016 using multi-temporal Landsat images. Through a comparative analysis, the authors concluded that 92% of these soils have become saltier over the studied period (referring to Fig. 6 in their paper). Although according to the literature, the predicted changes in soil salinity in arid and hyper-arid regions are feasible in the period considered in our study spanning over almost two centuries (1905–2100), physically constrained models are still required to evaluate the feasibility of occurrence of the conclusions obtained from our ML models. To conduct such a physically based analysis on a global scale, one would need detailed soil and environmental data to model precipitation, leaching events as well as in situ salt amount in the root zone on a global scale which is currently not available.

The changes predicted here do not agree with the global-scale predictions of Schofield et al.^57^ who estimated that Australia and western North America would be the areas with lower salinization potential in the 2070–2099 period, while they predicted a high potential for salinization in lands across Eastern Europe and Kazakhstan. In addition to the difference between the methodologies used for the projections of soil salinity, this discrepancy is due to various other reasons. For example, unlike the current study, Schofield et al.^57^ only used one GCM (HadCM3GGa), developed before 2000, to specify their salinization indicators. Furthermore, they estimated the future potential evapotranspiration as an empirical function of air temperature to calculate AI as an indicator of soil salinity, while we used total evapotranspiration derived from the more physically based GCMs.

The results of ML models are primarily based on the trends they capture from the input data used for training. Therefore, projected changes of EC_e_ in the hotspots of climate-induced soil salinization can be mainly attributed to the variations in spatio-temporal input data projected by GCMs. As mentioned before, precipitation frequency and evapotranspiration were the most influential spatio-temporal predictors for the predictions of the trained models. According to the analytical salt mass balance, higher evapotranspiration rate and precipitation with a lower frequency and intensity accumulate more salts in the root zone^66^. By the end of the century, an ensemble mean decrease in precipitation (under RCP 8.5) of up to 40% was reported by Giorgi et al^88^. for the southern hemisphere, particularly southern and western Australia, Namibia, and Brazil for the June–July–August months, which are also the salinization hotspots according to our results. Similarly, in the northern hemisphere, they predicted a more severe decrease in precipitation for Mexico, West Africa, and Mediterranean coasts for December–January–February. At smaller spatial scales, other studies projected an increase in the number and duration of drought events, higher potential and actual evapotranspiration, decreasing trends in frequency and intensity of precipitation, and in general drier conditions by the mid and end of the century.

Using 34 GCMs under the two different emission scenarios of RCP 4.5 and RCP 8.5, Shi et al.^89^ predicted that potential evapotranspiration tends to increase in south-eastern Australia. Likewise, using 22 CMIP5 models, a substantial increase in the number of warm temperature extremes and periods of dryness was projected by Alexander et al.^90^ for Australia, one of the predicted salinization hotspots in the current study. Similar trends for Australia were projected by Grose et al.^91^ by analysing the available CMIP6 multi-model ensemble. By analysis of 14 GCMs under the RCP 4.5 and RCP 8.5 future scenarios, a substantial decrease in precipitation during the summer (up to 1.5 mm day^−1^) is expected by Colorado‐Ruiz et al.^92^ in southern Mexico, also a projected salinization hotspots in the present study. A decrease in the frequency of precipitation during winter and spring in south-western United States is projected by Easterling et al.^93^, as also found in this study to be a hotspot. An increase in the number of consecutive dry days in west Sahara^94^ and actual evapotranspiration in arid areas across north-western China^95^ under the 1.5 °C and 2.0 °C global warming scenarios reported in the literature is congruent with the findings of the current study.

To conclude, lack of reliable predictive tools and data to assist land managers and policy makers for understanding the land cover dynamics is one of the main obstacles to long-term sustainable land and environment management. In the present study, we used legacy soil-profiles data and a set of purely spatial and spatio-temporal predictors to develop some predictive ML models for projection of the primary soil salinity (represented by electrical conductivity) as one of the major threats to the soil fertility, stability, and biodiversity in world drylands. Our analysis provides long-term gridded (at 0.5° spatial resolution) predictions of primary soil salinity change in drylands globally in response to projected key climatic drivers of soil salinity, which is currently missing in the soil and land management literature. In the face of projected future climatic uncertainties, the developed predictive models and generated data in the present investigation can help with decision-making regarding land and water resources management to recognize the hotspots of soil salinization, devise the necessary action plans, and implement those plans towards sustainable land and water resources management.

Under different GHG concentration trajectories, our predictions suggest that by the late 21th century the drylands areas of South America, southern Australia, Mexico, south-west United States, and South Africa are at the risk of higher soil salinity caused by climate change, compared to the reference period (1961–1990). In addition, increase in climate-induced soil salinity threatens the drylands of Spain, Morocco, and northern Algeria by the end of the century. On the other hand, our results project a decreasing trend in primary soil salinity of the drylands located in the northwest United States, the Horn of Africa, Eastern Europe, Turkmenistan, and west Kazakhstan, relative to the reference period. The reliability of the predictions made here are different: the projected soil salinities for the drylands located in North America and Australia are of the highest level of reliability while the drylands of central Asia, Middles East, and the Great Sahara have the highest uncertainty in predictions for soil salinity. Other zones such as India, South America, and South Africa are in the middle in terms of the reliability of predictions.

## Methods

In a previous study^33^, we developed tree-based two-part predictive ML models for determining annual surface (referring to top 30 cm of the soil) soil salinity and sodicity (represented by exchangeable sodium percentage) over the past four decades (1980–2018) at ~1 km^2^ spatial resolution on a global scale. In the present study, however, we aimed to predict the future dynamics of soil salinization up to the year 2100 under changing climate. In the present investigation, we focused on primary salinization and the trained tree-based ML models were only of regressive models. The next sections explain the details of the workflow for predicting soil salinity (EC_e_) including: (1) collection of the measured soil-salinity profiles, (2) collection and processing of salinity predictors, (3) exposing the salinity profiles and predictors data to ML models, training the models, and validation of the trained models, and (4) employing the trained models to project the spatio-temporal variation of the soil EC_e_ up to the year 2100 under different greenhouse gas (GHG) concentration trajectories. Finally, we discuss the accuracy of the trained models for prediction of EC_e_.

### Soil-salinity profiles

**Fig. 4:**
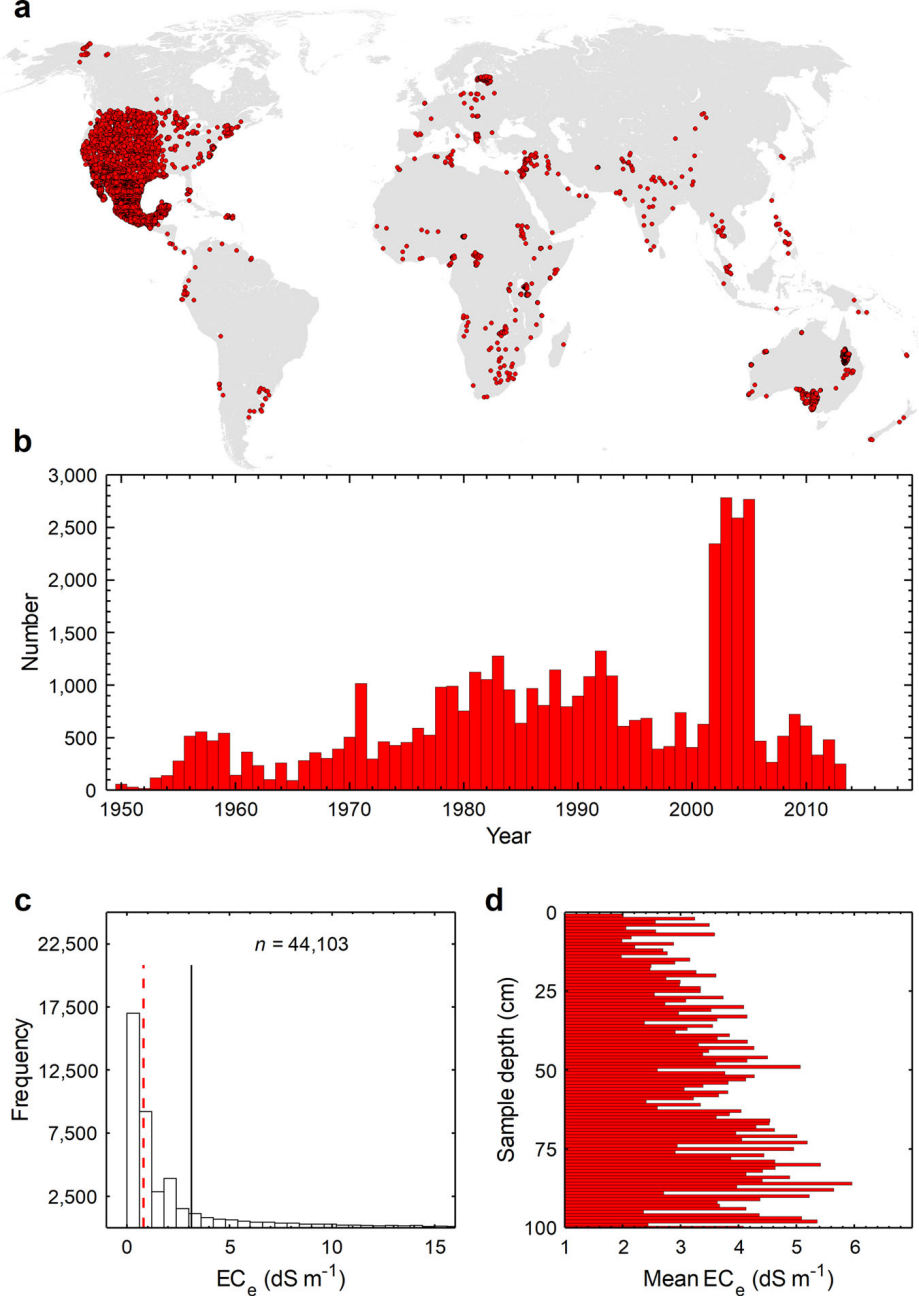
General properties of the EC_e_ profiles used for training the models. **a** spatial distribution of the soil salinity profiles used for model training and prediction of the soil salinity. Each profile includes one or more soil samples. **b** temporal distribution of the samples used for training the predictive models of soil salinity. Each bar shows the number of samples within one year. **c** frequency distribution of the measured values of EC_e_. The solid and dashed vertical lines represent the mean and median values, respectively. **d** average of the measured soil salinity values at 1 cm intervals to the depth of 1 m below the surface.

We obtained the geo-referenced soil profiles (points) with measured values of EC_e_ from the soil-profile dataset of World Soil Information Service (WoSIS)^96^. The spatial distribution of the profiles data used as an input into the ML models is presented in Fig. 4a. The WoSIS EC_e_ database includes 19,434 soil profiles and each individual profile (with a unique profile ID) may include one or more samples for various depths below the soil surface. The data cover the sampling period from 1950 to 2014. Since the date of sampling was an essential parameter in model training, we removed the EC_e_ profiles without sampling dates. This reduced the total number of EC_e_ samples from 73,517 to 59,649, with the number of samples per year shown in Fig. 4b. In addition, we dropped the soil EC_e_ profiles sampled from the croplands to remove the effects of human interventions from the analysis. As a result, a total 44,708 samples (11,517 profiles) remained in our analysis for model training and accuracy assessment.

Global land cover data provided by Earth-Observation Satellites before 1997 were scarce. Accordingly, we divided the profiles into two categories based on the date of sampling: before 1997 and after 1997. For the period before 1997, we identified the profiles located in croplands using the Global Land Cover Characteristics Database, Version 2.0 at ~1 km resolution^97^. Due to a lack of historical land cover data, we assumed that the land cover/land use did not change considerably before the 1980s. For profiles sampled after January 1997, however, we identified the samples/profiles located in croplands using land cover maps for years 2000, 2006, 2014, and 2018 with similar International Geosphere-Biosphere Programme (IGBP) land cover legend adopted from the MODIS Data Collection (MCD12Q1 and MCD12C1)^98^. We selected the IGBP land cover legend as it was available in both datasets. Each profile sampling date was attributed to the layer with the nearest year of acquisition. The MODIS land cover layers were first re-projected to the World Geodetic System (WGS 1984) spatial coordinates at 0.004° (~500 m) using the nearest neighbour method.

### Predictors

We used two types of predictor to train the models for predicting EC_e_ as the target variable: purely spatial and spatio-temporal. Purely spatial predictors included the land and soil attributes, which were relatively constant during the period of the analysis, while spatio-temporal predictors were the large-scale hydro-climatic variables derived from the output of selective GCMs. In total, 14 predictors were used, of which nine purely spatial and the rest spatio-temporal. The pre-processing details, projection, extent, and resolution of the predictors’ layers are summarized in Table 2. These predictors were primarily selected to represent the main factors affecting the salt balance in the root zone in non-irrigated soils^66^. In addition, we included in our model training additional soil formation factors, including topography and parent material (weathered rock or deposit from which the soil is formed)^96,99^.

**Table 2:** Purely spatial and spatio-temporal predictors used for model training and prediction of soil salinity.

Predictor	Pre-processing	Projection	Source layer spatial extent	Source (spatial resolution)
Sample upper depth (cm)^a^	–	–	–	Original soil dataset^96^ (Not applicable)
Sample lower depth (cm)^a^	–	–	–	ditto
Elevation (m)^a^	Resampling the original DEM to ~250 m resolution by the cubic convolution method	WGS 1984 Web Mercator (Auxiliary Sphere)	Left: −20,037,507.84 m Right: 20,037,507.90 m Bottom: −20,037,508.41 m Top: 20,037,508.34 m	World Elevation Terrain service Imagery Layer from Esri^103^ (0.25 m)
Slope (degrees)^a^	Resampling the original DEM to ~250 m resolution by the cubic convolution method and then calculating the slope using ArcGIS “slope” function	ditto	ditto	ditto
World Reference Base soil classes (120 classes)^a^	–	GCS WGS 1984	180W-180E, 62S-87.37 N	ISRIC-SoilGrids250^72^ (0.00208˚)
Soil clay content (%)^a^	Per-cell averages of five standard soil depths: 0, 15, 30, 60, and 100 cm were calculated using the trapezoidal rule and ArcGIS “cell statistics” tool	ditto	ditto	ditto
Field capacity (mm)^a^	Raster datasets for different continents were merged into a single global one	GCS WGS 1984	180W-180E, 56.49S-90N	Global Gridded Surfaces of Selected Soil Characteristics IGBP-DIS^101^ (0.00833˚)
Wilting point (mm)^a^	ditto	ditto	ditto	ditto
Effective plant rooting depth (m)^a^	The original dataset was geo-referenced to the GCS WGS 1984 coordinates system by the nearest neighbour method	GCS WGS 1984	180.25W-179.75E, 90.25S-89.75 N	Yang et al.^102^. (0.5˚)
Five-year moving average of annual precipitation frequency (day^−1^)^b^	Precipitation frequency (*λ*) was calculated by dividing the number of wet days (daily precipitation > 1 mm) by the total number of days of a year (*T*). Precipitation fluxes (kg m^−2^ s^−1^) were transformed to daily sums by multiplying by a factor of 86,400	GCS WGS 1984	180W-180E, 90S-90N	Global Circulation Models (GCMs) presented in Table 3
Five-year moving average of annual precipitation intensity (cm)^b^	Precipitation intensity was calculated by ≅ *αλT* = Annual accumulative precipitation, where *α* was the precipitation intensity, *λ* was precipitation frequency, and *T* was the total number of days of a year (365)	ditto	ditto	ditto
Five-year moving average of daily evapotranspiration (cm day^−1^)^b^	First an annual average was calculated from monthly evapotranspiration fluxes (kg m^−2^ s^−1^). Then the annual average flux was transformed to daily sum by multiplying by a factor of 8640	ditto	ditto	ditto
Five-year moving average of daily dry deposition rate of sea salts (mg day^−1^ m^−2^)^b^	First an annual average was calculated from monthly dry deposition rates (kg m^−2^ s^−1^). Then the annual average flux was transformed to daily sum by multiplying by a factor of 86,400	ditto	ditto	ditto
Five-year moving average of daily wet deposition rate of sea salts (mg day^−1^ m^−2^)^b^	First an annual average was calculated from monthly wet deposition rates (kg m^−2^ s^−1^). Then the annual average flux was transformed to daily sum by multiplying by a factor of 86,400	ditto	ditto	ditto

^a^Purely spatial predictor.

^b^Spatio-temporal predictor.

The purely spatial predictors comprised:

- soil classes based on the World Reference Base (WRB) classification^72,100^;
- soil texture represented by the percentage of clay content, obtained from the ISRIC global gridded soil information at ~250 m spatial resolution^72^;
- soil wilting point in mm^101^;
- soil field capacity in mm^101^;
- effective plant rooting depth in m^102^;
- topographic slope in degrees; and
- terrain elevation in m.

Slope and terrain elevation layers were derived from the World Elevation Terrain data adopted from ArcGIS Living Atlas of the World^103^ and were re-projected to the WGS 1984 coordinates system at 0.002° (~250 m) spatial resolution using the cubic convolution method. We filled the missing grid-cells (or cells with no data values) in purely spatial predictor layers with an average from the cells surrounding the missing grid-cell. We used a circle with a radius of four cells from the neighbouring cells to calculate the average and fill the data gap. All purely spatial predictors were assumed to be vertically constant. Raster processing was conducted in ArcGIS 10.7^104^. Then, we obtained the values of grid-cells of purely spatial predictors at the locations of EC_e_ profiles (Fig. 4a) to later train predictive models of soil salinity (see “model training for prediction of soil salinity”). The upper and lower depths of the measured EC_e_ samples derived from the original WoSIS database were the additional purely spatial predictors used for model training; these were introduced to account for the effect of depth on soil salinization processes.

The spatio-temporal predictors considered here were precipitation intensity, precipitation frequency, daily evapotranspiration, and sea salts wet and dry deposition rates (Table 2). To make predictions for future periods, we needed the projected values of the predictors. Therefore, we derived the values of spatio-temporal predictors from the outputs of the GCMs under different GHG concentration trajectories.

For training the models, we used the GCMs available in both CMIP5 and CMIP6 data projects to consider the uncertainty in GCMs predictions and to cover all available projections for dry and wet sea salt deposition rates. Additionally, this gave us the opportunity to analyse the differences between the CMIP5 and CMIP6 model outputs in terms of the derived predictors’ values and their effects on the projected soil salinity. The historical outputs of GCMs, including precipitation, evapotranspiration, and dry and wet deposition rates of sea salts, were used for training the predictive ML models (CMIP5: 1900–2005; CMIP6: 1900–2014). The projected outputs of GCMs for the same parameters were used to make future predictions of soil salinity (CMIP5: 2006–2100, CMIP6: 2015–2100). For the CMIP5 models, predictors were calculated based on the future projections forced by the RCP 4.5 and RCP 8.5 scenarios. Likewise, for the GCMs models of CMIP6, predictors were computed using future projections forced by RCP 4.5 and RCP 8.5 global forcing pathways for the Shared Socio-economic Pathways (SSP) 2 and 5, respectively. These medium (4.5) and high (8.5) radiative forcing pathways were chosen because they, respectively, represent the most plausible (or stabilization) and worst case scenarios of emissions by the end of the 21th century.

Since the total number of wet days and the total annual precipitation values were calculated from the daily precipitation fluxes, the GCMs with precipitation data at daily resolution were required. Additionally, not all of the available GCMs in the CMIP5 and CMIP6 projects had the dry and wet deposition rates of the sea salts. Accordingly, our analysis was narrowed down to a total of 16 GCMs outputs under different GHG concentration trajectories from both CMIP5 and CMIP6 projects. For the GCMs with different ensemble members (MIROC5 and CESM2-WACCM-gn, in particular), we computed an ensemble mean to avoid a bias in the results of final multi-GCM ensembles toward the GCMs with the higher number of participating ensemble members. In total, data of eight GCMs (seven with projections under RCP 4.5 and six GCMs with projections under RCP 8.5) and eight GCMs (with projections under both SSP 2-4.5 and SSP 5-8.5) were downloaded from the CMIP5 and CMIP6 data^105^, respectively. Details on the final chosen GCMs, their spatial resolution, and their used ensemble members are presented in Table 3.

**Table 3:** Global Circulation models (GCMs) used for calculation of the spatio-temporal predictors.

CMIP5^a^ and CMIP6 model names	Ensemble member(s)^b^	Scenario(s)	Spatial resolution (latitude × longitude)	Source
GISS-E2-H	r6i1p3	RCP 4.5	2° × 2.5°	NASA Goddard Institute for Space Studies^121^.
GISS-E2-R	r6i1p3	RCP 4.5	2° × 2.5°	NASA Goddard Institute for Space Studies^121^.
MIROC5	r1i1p1, r2i1p1, r3i1p1	RCP 4.5, RCP 8.5	1.4008° × 1.40625°	Atmosphere and Ocean Research Institute (The University of Tokyo), National Institute for Environmental Studies, and Japan Agency for Marine-Earth Science and Technology^122^.
MIROC-ESM-CHEM	r1i1p1	RCP 4.5, RCP 8.5	2.7906° × 2.8125°	Japan Agency for Marine-Earth Science and Technology, Atmosphere and Ocean Research Institute (The University of Tokyo), and National Institute for Environmental Studies^123^.
MIROC-ESM	r1i1p1	RCP 4.5, RCP 8.5	2.7906° × 2.8125°	Japan Agency for Marine-Earth Science and Technology, Atmosphere and Ocean Research Institute (The University of Tokyo), and National Institute for Environmental Studies^123^.
MRI-CGCM3	r1i1p1	RCP 4.5, RCP 8.5	1.12148° × 1.125°	Meteorological Research Institute^124^
NorESM1-M	r1i1p1	RCP 4.5, RCP 8.5	1.8947° × 2.5°	Norwegian Climate Centre^125^.
MRI-ESM1	r1i1p1	RCP 8.5	1.8947° × 2.5°	Meteorological Research Institute^126^
CESM2-WACCM-gn	r1i1p1f1, r2i1p1f1, r3i1p1f1	SSP 2-4.5, SSP 5-8.5	0.94240838° × 1.25°	Community Earth System Model Contributors^127^.
CNRM-ESM2-1-gr	r1i1p1f2	SSP 2-4.5, SSP 5-8.5	1.4003477° × 1.40625°	National Centre for Meteorological Research, Météo-France and CNRS laboratory^128^.
GFDL-ESM4-gr1	r1i1p1f1	SSP 2-4.5, SSP 5-8.5	1° × 1.25°	NOAA Geophysical Fluid Dynamics Laboratory^129^.
INM-CM4-8-gr1	r1i1p1f1	SSP 2-4.5, SSP 5-8.5	1.5° × 2°	Institute for Numerical Mathematics^130^.
INM-CM5-0-gr1	r1i1p1f1	SSP 2-4.5, SSP 5-8.5	1.5° × 2°	Institute for Numerical Mathematics^131^.
MIROC-ES2L-gn	r1i1p1f2	SSP 2-4.5, SSP 5-8.5	2.7889823° × 2.8125°	Atmosphere and Ocean Research Institute (The University of Tokyo), National Institute for Environmental Studies^132^.
MRI-ESM2-0-gn	r1i1p1f1	SSP 2-4.5, SSP 5-8.5	1.8645104° × 1.875°	Meteorological Research Institute^133^.
NorESM2-LM-gn	r1i1p1f1	SSP 2-4.5, SSP 5-8.5	1.8947368° × 2.5°	Norwegian Climate Centre^134^.

^a^Coupled Model Inter-comparison Project Phase 5.

^b^Indices define the ensemble member: “r” for realization, “i” for initialization, “p” for physics, and “f” for forcing. Ensemble members with four indices relate to CMIP6.

The original longitude values of netCDF files were set in the range −90° and 90°, referenced to the Greenwich Prime Meridian, to be in the same spatial extent as the purely spatial predictors. Then, using the bilinear interpolation method, all were interpolated to 0.5° × 0.5° WGS 1984 longitude-latitude regular grid to be able to generate multi-GCM ensemble from the outputs of our predictive models. Calculation of the spatio-temporal predictors and processing of the original netCDF files were conducted in the Climate Data Operators^106^ environment. The prepared netCDF data based on the outputs of GCMs were then converted to multi-band rasters, after which we obtained the values of spatio-temporal predictors at locations of EC_e_ profiles. These values combined with the values of purely spatial predictors were used to train the predictive models of soil salinity. It was not practical to use these spatio-temporal predictors at daily or monthly temporal resolutions because of strong intra/inter-annual fluctuations in these predictors^66^. Therefore, we used a 5-year moving average instead (as a smoother input) to better capture the effect of intra/inter-annual trends in these predictors on soil salinity variations. Finally, the 5-year moving averages of the spatio-temporal predictors were attributed to each observation of EC_e_ according to the year of sampling.

### Model training for prediction of soil salinity

The measured values of EC_e_ (target or response variable) and the values of each of the 14 predictors (each represented by one column of data), attributed to the measured values of EC_e_, were then imported to MATLAB for model training and validation. For each GCM, a separate matrix of data was prepared, with a total of 16 matrices. The WRB soil classes (as the only categorical predictor) were represented by a vector of positive integers that contained values assigned to different soil classes. The other 13 predictors were non-categorical represented by a set of real numbers. In spite of employing the method explained earlier for estimation of the missing cells in predictors’ layers, the values of some purely spatial predictors were still missing in the final imported matrices. Therefore, the corresponding EC_e_ values (each represented by a row of data) were eliminated and not used for model training. As a result, 1.28% of the sample rows were excluded from the analysis.

We applied MATLAB Statistics and ML toolbox (MATLAB, R2019b) for building and validating the predictive models of EC_e_. Here, we used an ensemble of regression trees for training and projecting the soil salinity based on the predictor datasets obtained from each of the 16 GCMs shown in Table 3. We chose tree-based models due to their relatively higher accuracy and computational speed compared to other ML algorithms^33,107^. Additionally, tree-based predictive models are highly flexible in mapping non-linear relations between the known predictors and known responses^108,109^ and are robust in handling outliers and collinearity concerns in environmental modelling^110,111^. The MATLAB built-in “fitrenemble” function was applied for training the regression ensembles.

The model hyperparameters, or parameters that should be set before launching the training process of a ML algorithm, were tuned using MATLAB automatic hyperparameter optimizer. These comprised ensemble aggregation method, number of learning cycles, learn rate, minimum leaf size, maximum number of splits, and number of variables to sample^107^. By varying the hyperparameters, the optimizer attempts to find a combination of their values which minimizes the “log (1 + cross-validation loss)”. Holdout cross-validation method (with 25% of data being held out) was used for optimization and the cross-validation loss was quantified using mean squared error. The optimizer used the Bayesian optimization algorithm with the “expected-improvement-per-second-plus” acquisition function. The maximum number of objective function evaluations was 100 since there was no notable decrease in the value of the observed minimum objective function after 100 evaluations. We repartitioned the cross-validation at every iteration and assumed the weight of all observation rows to be equal to one. We applied the log-transform to address the issue of right skewness in frequency distribution of the target variable; however, the log-transformation and back-transform of the predicted responses had a negligible impact on the accuracy of the trained modes.

The Bayesian optimization algorithm could return different results since its chosen acquisition function depends on the runtime of the objective function; the optimizer avoids the regions with extremely high runtimes. According to the non-reproducibility of the tuned set of hyperparameters, the model training and hyperparameter tuning jobs on each of 16 datasets were repeated 30 times (480 models in total). The maximum number of learning cycles was limited to 500 to keep the runtime for each training task below 10 min. High runtime and computational costs did not allow us to repeat the trainings more than 30 times. We accelerated the model training process by running the computations on a machine with 48 cores using the MATLAB Parallel Computing Toolbox. The goodness-of-fit of the trained models was evaluated by 10-fold cross-validation *R*^*2*^ (the extent of variation explained by the model^112^), root mean squared error (RMSE), mean absolute error (MAE), and Nash-Sutcliffe model efficiency coefficient (NSE^113^). Then we used the bias corrected and accelerated percentile method to calculate the 95% confidence intervals of the mean for each validation metric based on 1,000 bootstrap samples (with replacement) derived from the results of the 30 runs performed for each of the 16 datasets. Among the 30 trained models for each input training set, the one with the lowest RMSE was selected; we chose RMSE as it is more sensitive to large errors^114^. In total, 16 models remained in our analysis for soil salinity projections.

### Model implementation and soil salinity projection

We converted the world drylands layer delineated by the United Nations Environment Programme World Conservation Monitoring Centre^37^ to a raster layer at 0.5° spatial resolution for generation of a global soil base map of the drylands. From that layer, we constrained our analysis to areas with an AI ≤ 0.65 and masked out the grid-cells (pixels) with an AI > 0.65 to keep only the drylands in our analysis^37^. The remained raster had 24,045 grid-cells and we used it as the global soil base map of the drylands.

Similar to input training profiles data, we extracted the values of purely spatial and spatio-temporal predictors to the location of the base map grid-cells and then a 5-year moving average from the values of spatio-temporal predictors was computed. We applied the best chosen trained models to these new locations (cells) and the corresponding values of the predictors. As mentioned before, the degree of soil salinity and solute concentration change along the soil depth. Usage of the upper and lower depths of the samples as predictors in the model training enabled us to make predictions of EC_e_ at different depths below the soil surface. In this regard, the trained models can be considered as four-dimensional predictive models of soil salinity that make predictions for different longitudes, latitudes, depths, and times. For each pixel and each year, we predicted the values of soil salinity at five depths: 0, 10, 30, 60, and 100 cm. We used the trapezoidal rule to compute an average of the EC_e_ (dS m^−1^) to the depth of 1 m as follows:^72^

1 $${{{{{{\rm{EC}}}}}}}_{{{{{{\rm{e}}}}}},{{{{{\rm{ave}}}}}}}= \, \left[(10-0)\times ({{{{{{\rm{EC}}}}}}}_{{{{{{\rm{e}}}}}}}(10)\,+{{{{{{\rm{EC}}}}}}}_{{{{{{\rm{e}}}}}}}(0))+(30-10)\times ({{{{{{\rm{EC}}}}}}}_{{{{{{\rm{e}}}}}}}(30)+{{{{{{\rm{EC}}}}}}}_{{{{{{\rm{e}}}}}}}(10))+\ldots\right. \\ \,\left. (60-30)\,\times ({{{{{{\rm{EC}}}}}}}_{{{{{{\rm{e}}}}}}}(60)+{{{{{{\rm{EC}}}}}}}_{{{{{{\rm{e}}}}}}}(30))+(100-60)\,\times ({{{{{{\rm{EC}}}}}}}_{{{{{{\rm{e}}}}}}}(100)+{{{{{{\rm{EC}}}}}}}_{{{{{{\rm{e}}}}}}}(60))\right]/(100\,\times 2)$$

where EC_e_ is the predicted salinity at the corresponding depth. The outlier that is more than three scaled Median Absolute Deviations (MAD) away from the median of all predictions of a year were removed by the MATLAB “isoutlier” built-in function; this was the most robust method for removing outliers according to the user guide (see MATLAB “isoutlier” documentation for further details). In total, for each grid-cell of the global soil base map of the drylands, 197 predictions of EC_e_ were made in the period between 1904 and 2100 (one prediction for each year); since all spatio-temporal predictors are five-year moving averages, 1904 is the beginning of the period.

To compare the future state of the drylands soil salinity against the past conditions, we considered three time periods in our analysis: reference period (1961–1990), mid-term future (2031–2060), and long-term future (2071–2100). We used 30-year periods and 1961–1990 as the reference period based on the recommendations of the World Meteorological Organization for evaluations of the long-term changes in climatic variables^115^. Soil salinity predictions for years in the future periods were averaged and compered to the average of the predictions for years in the reference period.

We calculated the area of each grid-cell of the global soil base map of the drylands in the WGS 1984 spatial coordinates using the computer code presented in the Supplementary Information. We estimated the total annual area of salt-affected soils between 1904 and 2100 and then computed the annual percentage change in the area of those soils by dividing the total area at each year by the average area of salt-affected soils over the period. We assumed an average of 95 years would be enough to remove the potential noise introduced by the spatio-temporal predictors. We used global administrative areas dataset^116^ to estimate the total area of salt-affected soils at the national and continental levels. Numerical values representing the countries and continents were attributed to each cell of the base soil map.

### Accuracy assessment of the trained models

The results of hyperparameter tuning and the 10-fold cross-validation accuracy metrics of the best-fitted models are summarized in Supplementary Table 14. Supplementary Table 15 also presents the calculated lower and upper limits of 95% confidence intervals of the 10-fold cross-validation accuracy metrics, calculated for the trained models. For all 16 models, the MATLAB ensemble aggregation method of “LSBoost” was superior in fitting the models, compared to the “Bagged” method.

For the best-fitted models, the lowest *R*^*2*^ was 71.72% (with the 95% confidence intervals of 67.62–69.89%) related to the GISS-E2-R model, while the highest *R*^*2*^ between the measured and predicted values of EC_e_ was 73.95% (67.34–70.32%), calculated for the CNRM-ESM2-1 model (see Table 3 for the details of GCMs). For all 16 models, the average calculated 10-fold cross-validation *R*^*2*^ was 72.79%. Likewise, GISS-E2-R and CNRM-ESM2-1 were the models with the highest and lowest calculated values of RMSE, respectively. The average of 10-fold cross-validation RMSE for all 16 best-fitted models was 3.6, ranging from 3.52 (3.78–3.93) to 3.67 (3.76–3.95). This represents a normalized RMSE equal to ~6% (normalized to the observed range of the EC_e_ values).

**Fig. 5:**
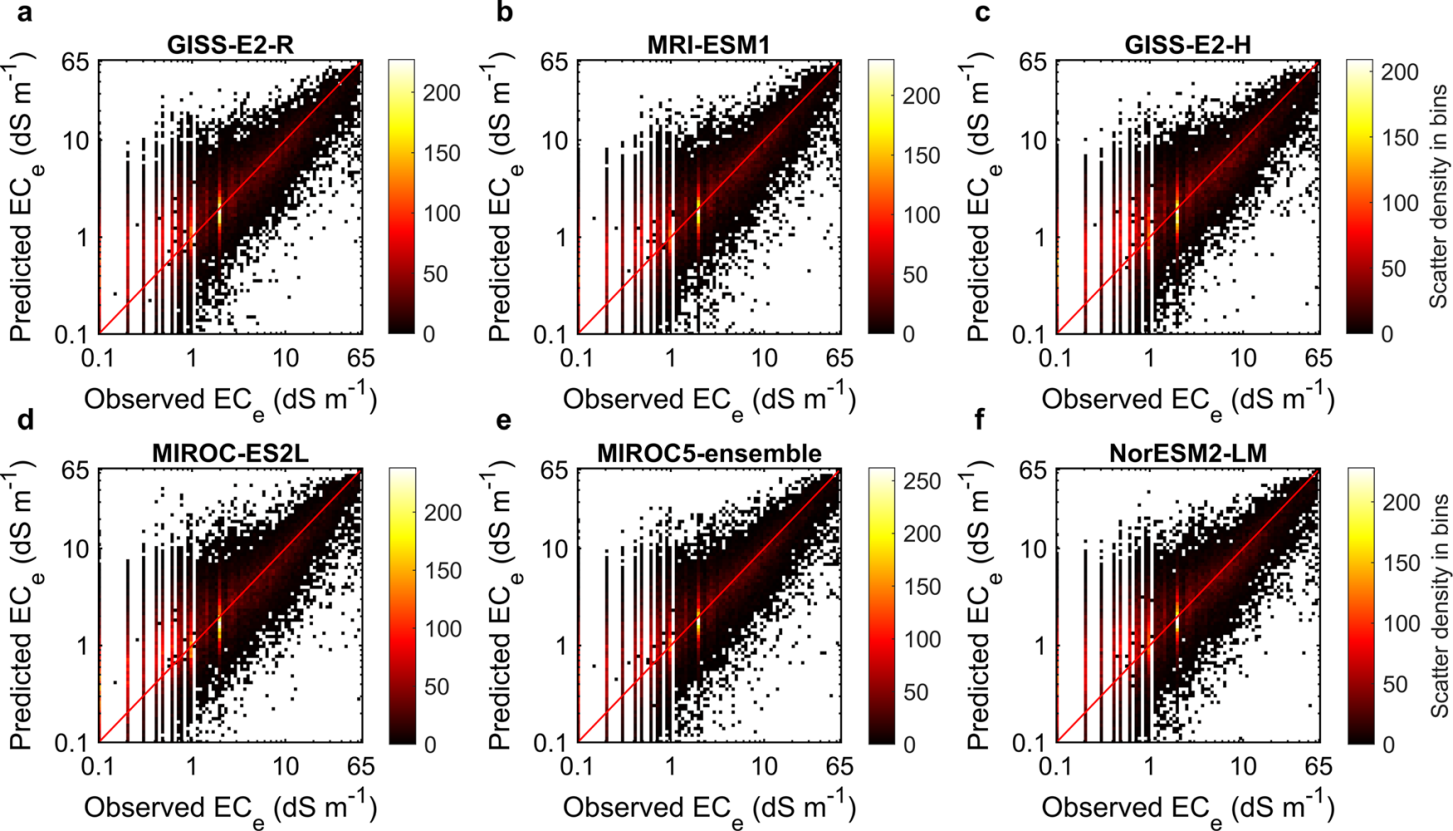
10-fold cross-validation plots for the six trained models with the highest root mean squared error (RMSE) values out of the final 16 best-fitted models. The RMSE decreases from **a**–**f**. The colour maps show the scatter density in each bin. The red lines represent the *y* = *x* line.

To understand better how well the best-fitted models predict the response values, the relation between the measured (values sampled from the soil profiles) and predicted values of EC_e_ is visualized in Fig. 5 via bin scatter plots. Taking a conservative approach, Fig. 5 shows only the validation plots for the six (out of the 16 best-fitted) models with the worst performance (i.e. with highest RMSE values). Predictions of the models are fairly concentrated around the *y* = *x* line, suggesting a good agreement of the modelled values with measured data. The accuracy of predictions increases with EC_e_ values, with a tendency for over-estimations for EC_e_ ≤ 1 dS m^−1^. Overall, the relatively high *R*^*2*^ (>70%) values indicate a satisfactory model fitting, particularly as such values are not common in digital soil mapping^117^.

Additionally, we evaluated the accuracy of the vertical prediction of the 16 best-fitted models, i.e. the prediction accuracy at various depths from the soil surface. To do so, we categorized the measured and predicted (by 10-fold cross-validation) values of EC_e_ into six bins of 0–20, 20–40, 40–60, 60–80, 80–100, and 100–200 cm based on an average from the lower and upper depths of the samples (each bin included its left edge); the bins edges were chosen so that the number of samples available for each bin stayed roughly equal and the deeper depths were not considered due to lack of data. The calculated *R*^*2*^ values for each bin and each of the 16 models are reported in Supplementary Table 16. The averages of the 16 models *R*^*2*^ values for the shallowest to deepest soil layers (bins) were 63.59%, 72.99% 77.39%, 77.31%, 79.59%, and 72.51%, respectively. These accuracies are in line with the reported *R*^*2*^ values of Taghizadeh-Mehrjardi et al.^28^ who developed separate regression tree-based models to predict soil salinity (78% for 0–15 cm soil layer). However, their analysis was purely spatial and was only focused on the saline soils located in a local area in central Iran (72,000 ha), while the current analysis projects the spatio-temporal variability in soil salinity on the global scale. We did not observe a decrease in predictive accuracy of the digital soil models at the higher depths reported in other studies, such as Malone et al.^117^ and Minasny et al.^118^.

**Fig. 6:**
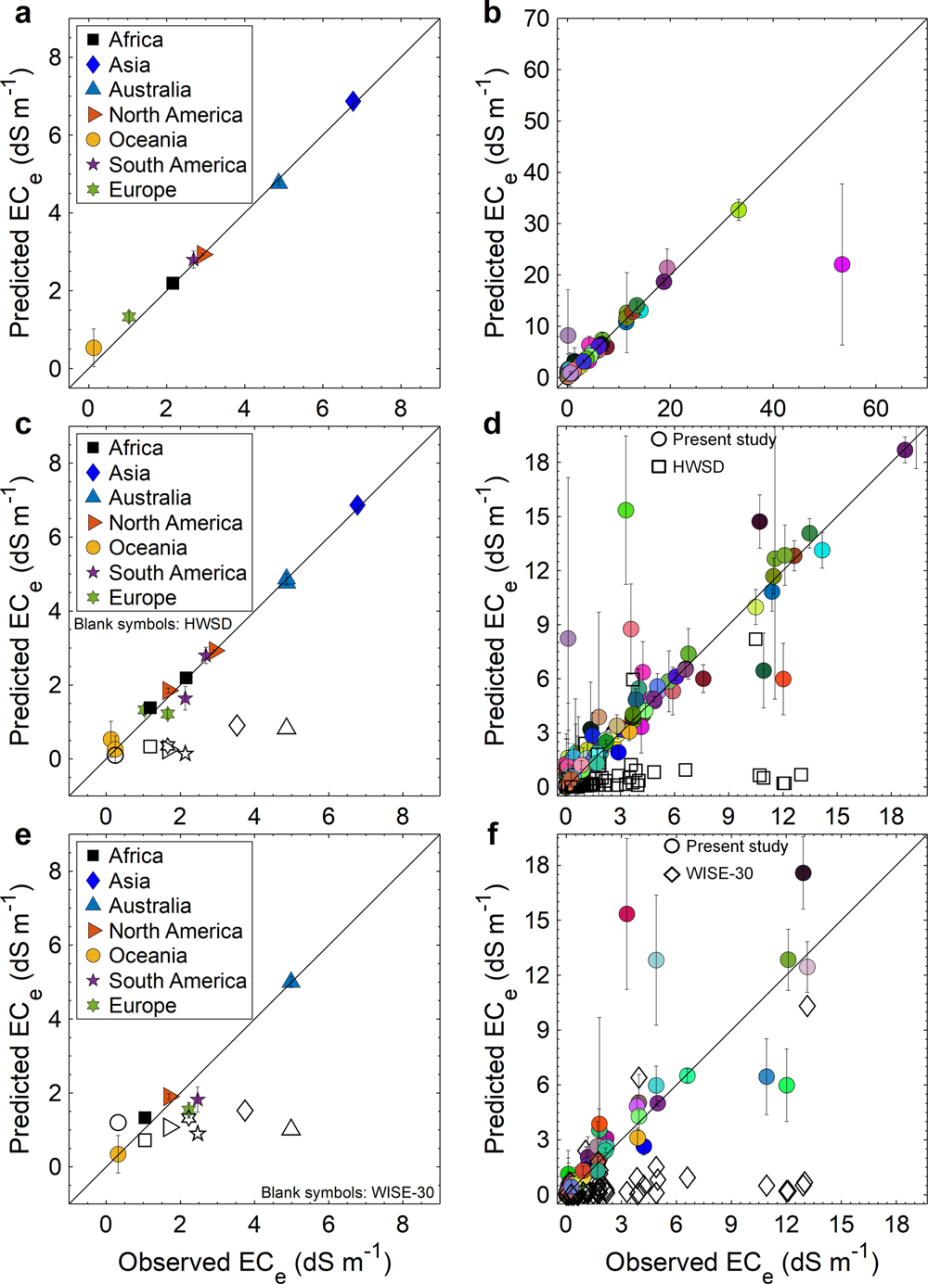
Comparison of the predicted values of soil salinity (EC_e_) in the present study and the measured values as well as the soil EC_e_ predicted in other datasets (i.e. HWSD and WISE-30) at the continental and country levels. **a**, **b** Average predicted values versus average measured values at the continental and country levels (87 countries), respectively. **c**, **d** Average of the surface (0–30 cm) salinity (EC_e_) values predicted by the present study and Harmonised World Soil Database (HWSD) versus the average of measured surface salinity at the continental and country levels (74 countries), respectively. **e**, **f** Average of the surface (0–20 cm) salinity predicted by the present study and WISE-30 (World Inventory of Soil Emission Potentials derived soil properties) dataset versus the average of measured surface salinity at the continental and country levels (71 countries), respectively. The error bars represent the minimum and maximum of average values calculated for the 29 models used in the study.

In addition to global accuracy assessment of the trained models, we evaluated the predictive power of the best-fitted models at the country and continental levels (Fig. 6a, b). We grouped the measured sample values of EC_e_ according to the continent or the country where the samples were acquired and compared the mean of each group with the mean of the 10-fold cross-validated predictions for each group. Only 87 countries had measured input profiles data of EC_e_ required for our analysis. At the country level, the *R*^*2*^ between the mean of predictions of the 16 models and the mean of measured values of EC_e_ was 80.41% while at the continental level, this value was 99.64%. The reason for such a high accuracy at the continental level is the high number of data points within each continent, which makes the predicted and estimated averages close to each other.

Similarly, we compared the predictions of our models with other available gridded datasets on soil EC_e_, including HWSD (Harmonised World Soil Database^10^) and WISE (World Inventory of Soil Emission Potentials) which derived soil properties on a 30 × 30 arc-seconds global grid (WISE-30; ref. ^119^), at the country and continental levels. Since these two datasets provide data for different soil layers (HWSD: two layers at 0–30 cm and 30–70 cm; WISE-30: seven layers, with five fixed depth intervals of 20 cm up to the depth of 100 cm and two 50 cm depth intervals between 100 and 200 cm), we only focused on surface measurements. For comparison with HWSD, any soil sample with the upper sample depth of 0 cm and a lower sample depth ≤30 was chosen as the surface measurement (a total of 8,995 samples) while for WISE-30, any EC_e_ sample with the lower sample depth of 20 cm was chosen as the surface measurement (a total of 7,535 samples).

At the location of each particular surface measurement, we predicted the soil salinity for 0–20 or 0–30 cm (depending on the target dataset for comparison) soil layers using the purely spatial and spatio-temporal values of predictors corresponding to the year of sampling of that particular surface measurement. Then we grouped the predictions and surface measurements based on the country and continent of sampling. At the country level, the *R*^2^ between the mean of our models predictions and the mean of surface measured values (0–30 cm) of EC_e_ for 74 countries was 68.55%, while this value for HWSD was 13.6%. At the continental level, these values were 91.48% and 74.98%, respectively (Fig. 6c, d). Compared to the WISE-30 predictions, the *R*^*2*^ between the mean of our models predictions and the mean of surface measured values (0–20 cm) of EC_e_ was 69.33% and 87.99% at the country (71 countries) and continental levels, respectively whereas the WISE-30 values were 17.22% and 5.53% (Fig. 6e, f). Although HWSD and WISE-30 datasets are purely spatial (they do not include information on the temporal variability of the soil salinity) and comparison is carried out with the same data used to train the ML models (as currently there are no other independent soil salinity datasets), comparing the predictions made by the models developed here against the predictions of those datasets can provide a better quantitative understanding of the improved predictive performance of our models.

### Model limitations, uncertainties, and perspectives for future research

ML models are one of the solutions suggested for time series projection challenges^120^. However, unlike the analytical models, ML models do not enable consideration of the mechanistic insights in the predictive algorithms of soil properties^72^. As mentioned earlier, no harmonized dataset is currently available quantifying the concentration of the soluble salts in salt-affected soils and, to a great extent, quantification of the severity of soil salinity in the field is limited to EC_e_ measurements. Provision of such dataset can be a baseline for developing more mechanistic and physically constrained approaches in projections of soil salinity. Although very challenging, partly due to the lack of the required environmental and soil data, development of root zone salt-budget models for projecting large-scale soil salinity driven by groundwater table, irrigation practices, and sea level rise is an important area for future research.

Captured trends and projections in this study depend on the input data used for training the models. Inconsistency in accuracy and methods applied by different laboratories for measuring soil properties can negatively impact the trends captured by the trained models. As we go towards the past, the number of available samples and their accuracy decreases (Fig. 4b); this in turn may influence the validation procedures applied to the predictions made by ML models^72^. It may also generate predictions biased towards the recent periods when more data samples are available. Additionally, more care should be given to application of the predictions made here at locations underrepresented by input data for training the ML models. In the current study, the majority of soil profiles used for training were sampled from North America and Australia due to a greater data availability. Thus, there is a possibility that the results are biased towards the soil and hydro-climatic conditions of these two continents. One solution to address this issue can be to develop more regional ML models; yet, this is challenging in the locations with the low number of sample data. Decrease in the number of available input data reduces the efficiency of the model training, resulting in less accurate and unsatisfactory validation outcomes. More updated and geographically scattered profile data are required in future studies to address the issue of inconsistency in the legacy soil-profile data. Although our analysis is an estimation of a relative change (relative to the reference period) in primary soil salinity and biases in GCMs outputs are not significant, application of reanalysis data for the reference historical period may address the biases issue in GCMs.

More importantly, the extent of uncertainty in the predictors used for training the models is not spatially constant. All the predictors used here are large-scale estimations of other models, which inherently include some degrees of uncertainty. Particularly, purely spatial predictors including the wilting point, field capacity, and effective plant rooting depth, are less certain in large deserts where observations are scarce for tuning and validation of the models. One way to address this issue is to provide spatially explicit maps of uncertainty for the predictions of the ML algorithms. However, this needs spatially explicit uncertainty maps of the predictors or their probability distributions. In the case of our study, such data were not available for the predictors. Additionally, ML algorithms are highly computationally demanding and estimation of the outputs uncertainty ranges by methods such as Monte Carlo simulations was not feasible by our computational resources (assuming hypothetical distributions of uncertainty in the predictors and input profiles data). Thus, we did not quantify the posterior distribution and uncertainty of the predictions and instead we estimated the global accuracy of the projected results via the 10-fold cross-validation method. A less computationally intensive framework is needed in the future for provision of the spatially explicit estimations of uncertainties in outputs of the ML models. Furthermore, comparison of our predictions accuracy with HWSD and WISE-30 datasets was based on the data used here for ML training and more independent datasets of soil salinity are required to benchmark our models performance against previous datasets/models of soil salinity.

The number of GCMs with projected wet and dry sea salt deposition rates (which are also necessary for mechanistic approaches) were rather limited in both CMIP5 and CMIP6 data projects. More ensemble members could improve the certainty of the projected soil salinity. Furthermore, the spatial resolution of our salinity projections was relatively coarse (0.5°); although the purely spatial predictors were of the adequate resolution, there was no point in prediction of the soil salinity values at finer resolutions since the spatio-temporal resolution of the GCMs grids was roughly between 1 and 3°. Such issues might be addressed with improvement of the spatial resolution of GCMs and the number of GCMs with sea salt aerosols projections in upcoming years.

## Supplementary information

Supplementary Information

## Data Availability

Data generated in this study including input data for training the predictive models, objects of the predictive models, annual predictions made by the models for each location, and spatially explicit maps quantifying the change in predicted soil salinity in the mid- (2031–2060) and long-term futures (2071–2100), relative to the reference period (1961–1990) have been deposited in the “figshare” database, freely available at 10.6084/m9.figshare.14548947.

## Code availability

Computer codes required for regeneration of the main results presented in this paper can be found in Supplementary Information appendix (computer codes section).
