# Supplementary material for: Global predictions of primary soil salinization under changing climate in the 21st century
Source: Nat Commun. 2021 Nov 18;12:6663. doi: 10.1038/s41467-021-26907-3 (PMC8602669; doi:10.1038/s41467-021-26907-3)
Supplement: Supplementary file 1 — Supplementary Information [file 41467_2021_26907_MOESM1_ESM.pdf]

Supplementary Information

Climate change and primary soil salinization: A global scale perspective for the 21st century

Amirhossein Hassani○ (1), Adisa Azapagic* (1), and Nima Shokri▪ (2)

(1) Department of Chemical Engineering and Analytical Science, The University of Manchester, Sackville Street, Manchester M13 9PL, UK

(2) Institute of Geo-Hydroinformatics, Hamburg University of Technology, Am Schwarzenberg-Campus 3 (E), 21073 Hamburg, Germany

○amirhossein.hassani@manchester.ac.uk, *adisa.azapagic@manchester.ac.uk, and ▪ nima.shokri@tuhh.de

Supplementary Table 1: Predictor importance (percentage) in the final 16 best fitted models.

**Model name**	**Sample upper depth**	**Sample lower depth**	**WRB**	**Clay content**	**Elevation**	**Slope**	**Field capacity**	**Wilting point**	**Effective plant rooting depth**	**dryss**[a]	**wetss**[b]	**evspsbl**[c]	**Pr-fre**[d]	**Pr-int**[e]
CMIP5 models														
GISS-E2-H	3.31	4.03	16.43	8.56	7.43	7.58	4.32	4.24	5.42	3.30	10.77	6.20	11.74	6.66
GISS-E2-R	3.57	3.57	14.65	7.23	5.68	7.18	3.91	3.13	5.00	4.06	5.69	10.35	16.47	9.50
MIROC5-ensemble	4.37	4.56	12.61	10.58	6.48	7.60	3.85	3.70	5.47	3.99	7.00	10.10	13.70	5.99
MIROC-ESM-CHEM	5.36	4.70	14.14	8.72	10.77	6.18	3.45	4.84	5.02	6.39	5.47	8.12	12.43	4.39
MIROC-ESM	5.54	5.86	12.95	7.71	11.26	6.50	3.73	5.00	4.81	5.98	6.10	9.01	10.83	4.72
MRI-CGCM3	4.26	4.05	13.91	8.71	6.10	6.77	3.92	3.83	5.80	5.70	5.14	11.17	14.14	6.49
MRI-ESM1	4.18	4.32	14.19	9.52	6.00	7.11	3.79	3.69	5.61	4.93	5.10	10.23	15.48	5.85
NorESM1-M	5.36	4.96	11.38	7.31	6.18	8.11	3.95	3.53	5.95	4.60	4.69	13.46	14.94	5.57
CMIP6 models														
CESM2-WACCM-ensemble	4.91	4.84	11.05	7.35	6.60	7.24	4.08	3.45	5.57	3.71	10.93	9.74	13.97	6.56
CNRM-ESM2-1	5.39	4.75	12.83	7.95	6.20	7.22	3.70	3.40	6.08	5.52	5.28	9.00	17.40	5.27
GFDL-ESM4	5.51	5.22	11.79	8.80	7.72	6.52	3.71	4.88	3.68	6.46	5.84	8.23	16.77	4.88
INM-CM4-8-	4.82	5.61	11.69	7.37	6.74	7.75	4.40	3.61	5.75	4.51	10.40	8.83	12.80	5.73
INM-CM5-0	3.78	4.61	13.79	7.61	6.74	7.91	4.07	3.57	5.71	3.65	9.02	8.12	15.87	5.54
MIROC-ES2L	5.14	4.85	14.11	9.15	11.01	5.63	3.87	1.51	10.28	4.59	9.11	6.55	7.19	7.00
MRI-ESM2-0	5.54	5.15	10.92	6.47	10.71	6.43	4.11	4.54	4.31	6.06	5.12	8.19	17.08	5.36
NorESM2-LM	4.39	4.29	12.72	7.84	7.66	7.86	5.11	4.12	2.78	7.68	6.31	10.82	13.33	5.11
Average	4.71	4.71	13.07	8.18	7.71	7.10	4.00	3.82	5.45	5.07	7.00	9.26	14.01	5.91

[a] Five-year moving average of daily dry deposition rate of sea salts.
[b] Five-year moving average of daily wet deposition rate of sea salts.
[c] Five-year moving average of daily evapotranspiration.
[d] Five-year moving average of annual precipitation frequency.
[e] Five-year moving average of annual precipitation intensity.

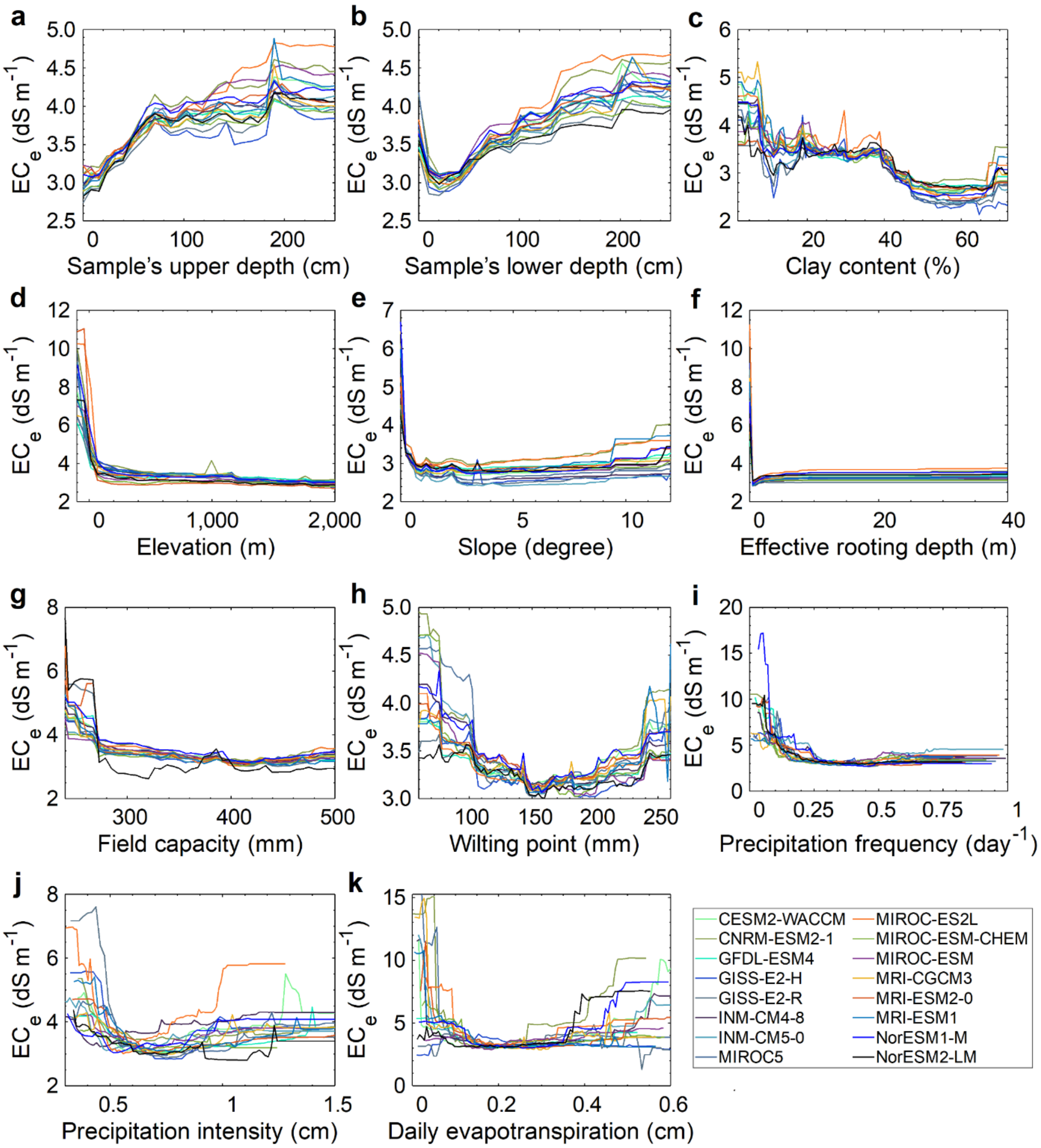

Supplementary Fig. 1: The relation between predicted soil salinity level (represented by EC_e) and the predictors used for training each of 16 predictive models of soil salinity. a to **h**, purely spatial predictors. **i** to **k**, long-term averages of the spatio-temporal predictors. The legend indicates the name of the corresponding original Global Circulation Models (GCMs) outputs.

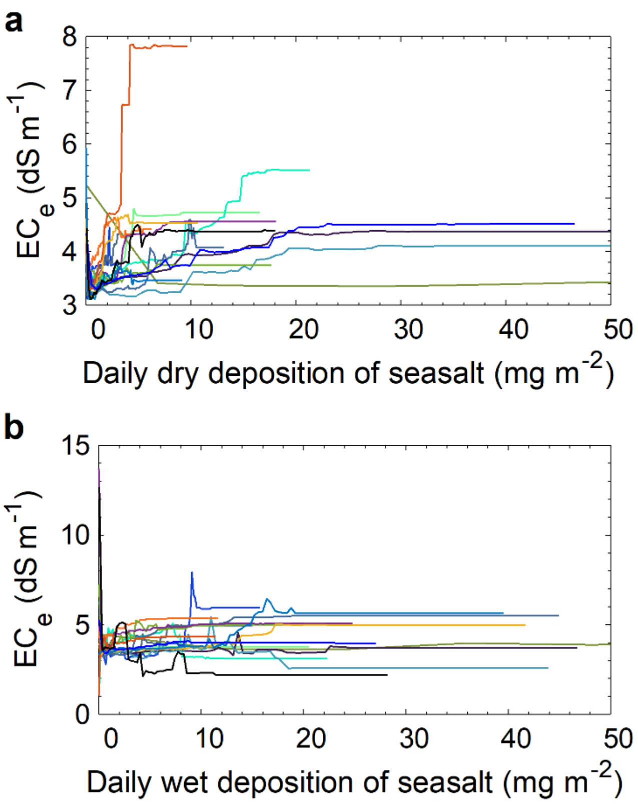

Supplementary Fig. 2: The relation between predicted soil salinity level (represented by EC_e) and long-term average of daily deposition rates of sea salts used as predictors for training each of 16 predictive models of soil salinity. **a**, dry deposition rate. **b**, wet deposition rate. Each line and colour is related to a model trained based on the original Global Circulation Models (GCMs) outputs (see Supplementary Fig. **1** for the full name of models and corresponding colours).

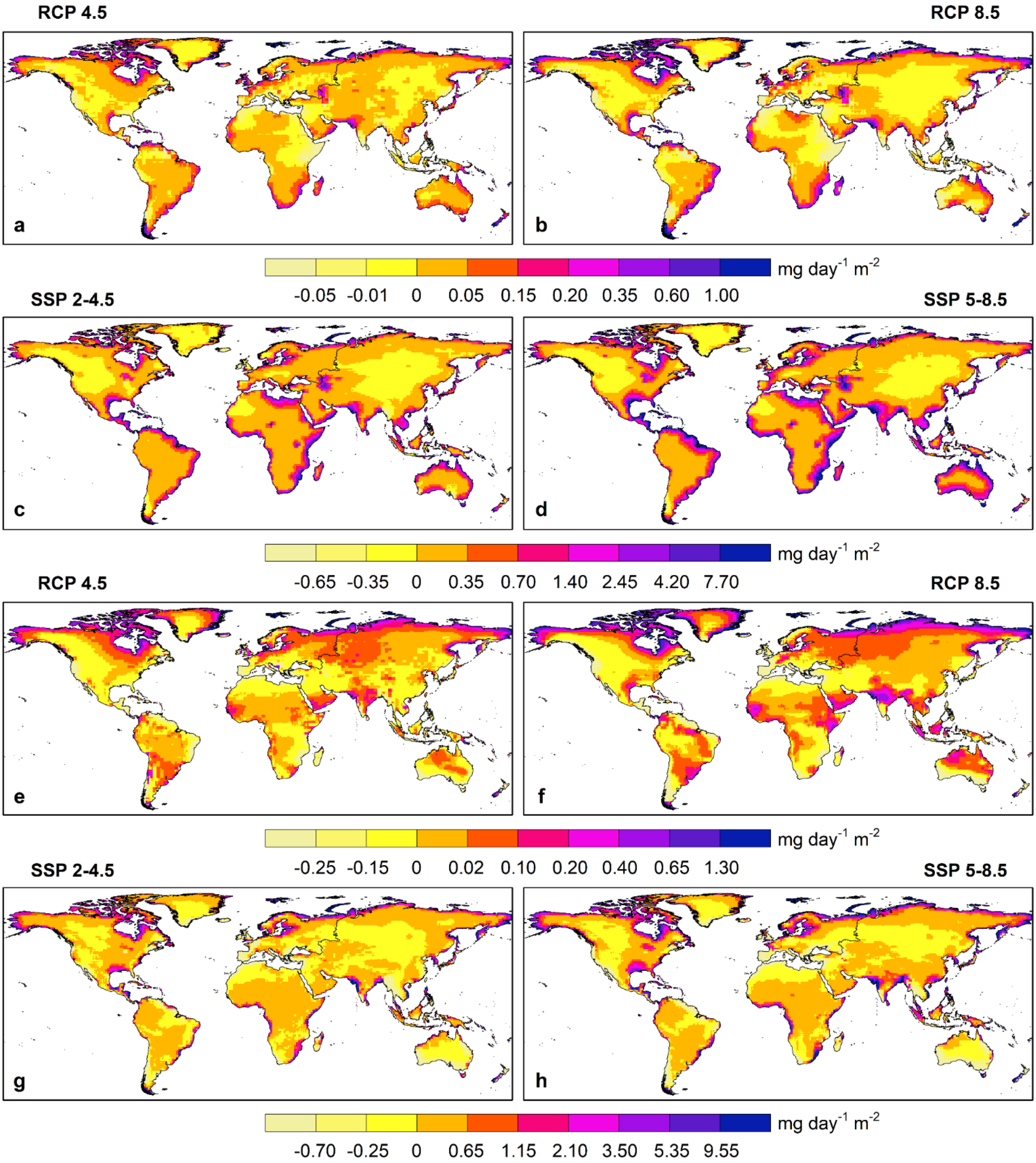

Supplementary Fig. 3: Multi-model ensemble mean of the change in deposition rates of sea salts by the end of the century (2071 - 2100), relative to the reference period (1961 - 1990) under different greenhouse gas concentration trajectories. a to **d**, change in the five-year moving average of daily dry deposition rate of sea salts. **e** to **h**, change in the five-year moving average of daily wet deposition rate of sea salts. Positive values indicate an increase in the deposition rate while negative values are indicative of a decrease.

Supplementary Table 2: Computed statistics based on the multi-model ensemble mean of the predicted gird-cell level relative change in soil salinity (EC_e) in the mid-term future (2031 - 2060) relative to the reference period (1961 - 1990) for the 30 countries with the highest number of dryland grid-cells in our analysis under RCP 4.5 greenhouse gas concentration trajectory.

RCP 4.5 mid-term future (%)								
**Country**	**Mean**	**Meanci-low[a]**	**Meanci-up[b]**	**Sem[c]**	**Min**	**Max**	**STDEV[d]**	**Variance**
Namibia	6.55	5.60	7.50	0.48	-17.35	26.20	7.92	62.65
Brazil	4.86	3.69	6.04	0.60	-19.76	27.86	10.96	120.02
Mexico	4.22	3.31	5.14	0.46	-22.39	27.98	10.12	102.42
South Africa	4.21	3.34	5.08	0.44	-18.26	28.05	7.76	60.28
Niger	3.67	2.62	4.72	0.53	-25.84	27.98	9.94	98.78
Chad	3.19	2.09	4.28	0.56	-25.22	27.29	10.84	117.59
United States	3.17	2.66	3.67	0.26	-22.90	27.42	10.05	100.99
Mauritania	3.01	1.93	4.10	0.55	-25.78	27.30	9.80	95.97
Mali	2.40	1.18	3.62	0.62	-26.28	28.07	11.94	142.48
Argentina	2.12	1.43	2.82	0.35	-22.33	27.88	8.67	75.13
Pakistan	1.94	1.15	2.73	0.40	-23.64	21.93	6.63	43.96
China	1.60	1.17	2.04	0.22	-25.22	28.10	8.76	76.70
Libya	1.24	0.46	2.03	0.40	-22.54	26.50	9.30	86.52
Ethiopia	0.89	-0.08	1.86	0.49	-18.04	26.62	7.26	52.78
Botswana	0.57	-0.52	1.67	0.56	-20.02	28.01	7.97	63.50
Russia	0.57	0.13	1.01	0.22	-26.51	27.89	10.00	99.96
India	0.43	-0.34	1.21	0.40	-26.43	28.00	9.89	97.89
Australia	0.43	0.15	0.71	0.14	-25.55	28.08	7.00	49.06
Kazakhstan	0.39	-0.14	0.91	0.27	-25.83	28.07	9.31	86.71
Afghanistan	0.17	-0.44	0.79	0.31	-10.56	18.33	4.72	22.24
Canada	-0.19	-0.66	0.28	0.24	-22.69	24.51	6.92	47.93
Iran	-0.19	-0.76	0.38	0.29	-18.59	25.36	7.01	49.17
Bolivia	-0.69	-1.98	0.60	0.65	-24.65	25.54	9.18	84.29
Somalia	-0.80	-2.10	0.51	0.66	-26.90	27.68	9.41	88.57
Sudan	-1.72	-2.52	-0.93	0.40	-25.24	27.52	9.92	98.44
Turkey	-1.96	-3.12	-0.80	0.59	-20.59	27.02	9.24	85.45
Algeria	-3.88	-4.57	-3.18	0.35	-25.40	27.35	9.88	97.54
Egypt	-4.56	-5.22	-3.91	0.33	-18.03	25.50	6.40	41.00
Mongolia	-5.50	-6.61	-4.39	0.56	-26.74	25.34	11.96	143.02
Saudi Arabia	-5.81	-6.36	-5.27	0.28	-24.93	26.26	7.17	51.45

[a] Lower limit of the 95% confidence interval for the mean.
[b] Upper limit of the 95% confidence interval for the mean.
[c] Standard error of the mean.
[d] Standard Deviation.

Supplementary Table 3: Computed statistics based on the multi-model ensemble mean of the predicted gird-cell level relative change in soil salinity (EC_e) in the mid-term future (2031 - 2060) relative to the reference period (1961 - 1990) for the 30 countries with the highest number of dryland grid-cells in our analysis under RCP 8.5 greenhouse gas concentration trajectory.

RCP 8.5 mid-term future (%)								
**Country**	**Mean**	**Meanci-low**	**Meanci-up**	**Sem**	**Min**	**Max**	**STDEV**	**Variance**
Namibia	7.15	6.31	7.99	0.43	-8.33	23.47	6.89	47.41
South Africa	5.36	4.37	6.35	0.50	-22.73	24.01	8.52	72.53
Brazil	4.76	3.77	5.76	0.51	-17.06	24.16	9.25	85.54
Botswana	3.38	2.29	4.48	0.56	-20.60	23.91	7.88	62.14
Mauritania	2.83	1.60	4.07	0.63	-20.95	23.95	10.88	118.34
United States	2.44	2.03	2.85	0.21	-24.52	24.01	8.16	66.54
Argentina	2.36	1.78	2.93	0.29	-18.92	23.41	7.21	52.00
Australia	2.19	1.96	2.42	0.12	-15.52	24.08	5.66	32.05
Niger	1.72	0.63	2.81	0.55	-24.22	23.50	10.02	100.41
Bolivia	1.56	0.55	2.57	0.51	-18.60	23.48	7.48	56.02
Mali	0.58	-0.47	1.63	0.54	-23.40	23.93	10.22	104.49
Mexico	0.51	-0.34	1.35	0.43	-22.47	22.93	9.37	87.71
Chad	0.33	-0.85	1.50	0.60	-24.92	23.89	11.33	128.32
Pakistan	-0.33	-1.02	0.35	0.35	-12.94	22.13	5.78	33.36
Libya	-0.63	-1.28	0.02	0.33	-18.39	23.93	7.77	60.37
Somalia	-0.94	-1.99	0.11	0.53	-18.35	23.68	7.64	58.41
China	-0.96	-1.44	-0.47	0.25	-24.84	23.81	9.47	89.69
Canada	-1.50	-1.94	-1.05	0.23	-21.11	23.34	6.61	43.67
India	-1.68	-2.23	-1.14	0.28	-23.26	23.84	7.11	50.48
Russia	-2.31	-2.65	-1.98	0.17	-23.66	23.24	7.66	58.65
Afghanistan	-2.39	-3.00	-1.77	0.31	-14.12	10.06	4.78	22.89
Kazakhstan	-2.55	-2.92	-2.18	0.19	-22.87	19.98	6.69	44.71
Sudan	-2.56	-3.21	-1.90	0.33	-21.44	24.12	8.30	68.92
Ethiopia	-2.59	-3.58	-1.60	0.50	-22.11	19.80	7.46	55.69
Turkey	-3.74	-4.72	-2.76	0.50	-23.70	22.09	7.89	62.29
Egypt	-4.59	-5.23	-3.94	0.33	-15.56	22.20	6.27	39.31
Algeria	-4.69	-5.33	-4.04	0.33	-20.08	24.11	8.99	80.79
Iran	-4.89	-5.44	-4.33	0.28	-23.86	23.11	6.84	46.84
Mongolia	-5.99	-7.00	-4.97	0.52	-24.58	23.89	10.93	119.39
Saudi Arabia	-6.92	-7.30	-6.54	0.19	-24.84	12.93	5.04	25.40

Supplementary Table 4: Computed statistics based on the multi-model ensemble mean of the predicted gird-cell level relative change in soil salinity (EC_e) in the mid-term future (2031 - 2060) relative to the reference period (1961 - 1990) for the 30 countries with the highest number of dryland grid-cells in our analysis under SSP 2-4.5 greenhouse gas concentration trajectory.

SSP 2-4.5 mid-term future (%)								
**Country**	**Mean**	**Meanci-low**	**Meanci-up**	**Sem**	**Min**	**Max**	**STDEV**	**Variance**
Botswana	8.97	8.09	9.85	0.45	-3.03	22.05	6.11	37.29
Mongolia	7.48	6.37	8.58	0.56	-10.61	21.78	8.17	66.79
South Africa	6.24	5.46	7.02	0.40	-16.35	22.09	6.93	48.08
Brazil	5.93	5.15	6.70	0.39	-18.27	22.02	7.41	54.91
Australia	5.77	5.52	6.01	0.12	-14.28	22.05	5.96	35.47
Namibia	5.44	4.73	6.16	0.36	-11.54	21.98	5.96	35.55
Mauritania	2.32	1.83	2.80	0.25	-12.72	18.28	4.75	22.58
Bolivia	1.19	0.30	2.07	0.45	-19.41	19.69	6.58	43.31
China	0.75	0.33	1.18	0.22	-21.58	22.08	8.08	65.36
Mexico	0.70	0.13	1.26	0.29	-17.72	20.89	6.38	40.70
Algeria	0.30	-0.03	0.64	0.17	-10.70	19.37	4.89	23.90
Libya	0.14	-0.31	0.59	0.23	-22.20	20.91	5.50	30.23
Mali	0.03	-0.50	0.56	0.27	-21.86	17.93	5.46	29.84
Egypt	-0.24	-0.66	0.18	0.21	-9.57	15.81	4.07	16.59
Sudan	-0.59	-1.10	-0.07	0.26	-22.18	20.73	6.59	43.38
Saudi Arabia	-0.72	-1.24	-0.19	0.27	-18.86	18.83	7.00	48.99
Argentina	-0.92	-1.38	-0.47	0.23	-21.72	22.09	5.70	32.45
Afghanistan	-1.16	-1.85	-0.47	0.35	-16.47	19.73	5.36	28.77
Turkey	-1.61	-2.36	-0.86	0.38	-17.12	17.25	6.03	36.40
United States	-2.24	-2.62	-1.86	0.19	-21.30	22.01	7.83	61.23
Kazakhstan	-2.29	-2.65	-1.94	0.18	-21.53	22.04	6.43	41.32
Pakistan	-2.42	-3.28	-1.56	0.44	-19.23	13.76	7.29	53.10
Canada	-2.53	-2.86	-2.19	0.17	-20.11	12.00	4.98	24.77
Chad	-3.21	-3.90	-2.52	0.35	-22.31	17.93	7.02	49.21
Iran	-3.31	-3.80	-2.82	0.25	-18.82	22.06	6.08	36.97
Ethiopia	-3.78	-4.50	-3.05	0.37	-17.31	10.74	5.48	30.06
India	-4.22	-4.78	-3.67	0.28	-22.18	21.48	7.20	51.86
Niger	-4.58	-5.32	-3.84	0.38	-21.82	19.33	7.49	56.12
Russia	-5.50	-5.79	-5.20	0.15	-22.19	21.28	6.58	43.33
Somalia	-6.82	-7.66	-5.99	0.42	-20.25	19.53	6.13	37.55

Supplementary Table 5: Computed statistics based on the multi-model ensemble mean of the predicted gird-cell level relative change in soil salinity (EC_e) in the mid-term future (2031 - 2060) relative to the reference period (1961 - 1990) for the 30 countries with the highest number of dryland grid-cells in our analysis under SSP 5-8.5 greenhouse gas concentration trajectory.

SSP 5-8.5 mid-term future (%)								
**Country**	**Mean**	**Meanci-low**	**Meanci-up**	**Sem**	**Min**	**Max**	**STDEV**	**Variance**
Botswana	9.53	8.56	10.50	0.49	-20.40	24.33	6.96	48.40
South Africa	8.54	7.70	9.39	0.43	-12.86	24.36	7.40	54.75
Brazil	8.00	7.12	8.87	0.45	-15.79	24.79	8.11	65.80
Namibia	6.62	5.77	7.47	0.43	-9.47	24.65	7.06	49.90
Mongolia	5.81	4.36	7.27	0.74	-19.68	24.29	10.91	118.96
Australia	5.75	5.51	5.99	0.12	-16.52	24.76	5.98	35.70
Mexico	3.55	2.86	4.25	0.35	-14.70	24.38	7.73	59.81
Mauritania	3.23	2.67	3.78	0.28	-10.70	24.69	5.40	29.14
Mali	1.23	0.58	1.88	0.33	-20.94	23.08	6.67	44.47
Bolivia	1.13	0.10	2.16	0.52	-21.25	22.92	7.64	58.31
Libya	0.80	0.32	1.27	0.24	-16.78	22.69	5.75	33.05
Algeria	0.69	0.24	1.14	0.23	-18.33	24.17	6.58	43.25
Afghanistan	0.21	-0.56	0.99	0.39	-13.76	21.02	6.01	36.16
China	0.09	-0.33	0.51	0.21	-22.80	24.78	8.20	67.26
Egypt	-0.05	-0.56	0.46	0.26	-11.99	22.86	4.98	24.85
Argentina	-0.60	-1.06	-0.14	0.24	-18.37	23.22	5.82	33.82
Sudan	-0.97	-1.63	-0.30	0.34	-22.45	24.63	8.47	71.79
United States	-1.08	-1.56	-0.61	0.24	-24.89	24.72	9.71	94.19
Turkey	-1.89	-2.89	-0.88	0.51	-21.19	23.61	8.02	64.39
Pakistan	-2.34	-3.17	-1.51	0.42	-20.40	24.14	7.01	49.08
Iran	-2.70	-3.18	-2.22	0.25	-16.43	24.31	5.94	35.29
Canada	-2.90	-3.26	-2.54	0.18	-21.20	16.10	5.39	29.06
Kazakhstan	-3.06	-3.41	-2.71	0.18	-21.43	24.55	6.27	39.31
India	-3.18	-3.79	-2.56	0.31	-24.23	24.80	7.96	63.30
Ethiopia	-3.26	-4.34	-2.19	0.55	-23.83	24.41	8.09	65.38
Saudi Arabia	-3.70	-4.22	-3.18	0.26	-22.36	23.92	6.85	46.93
Chad	-4.00	-4.83	-3.17	0.42	-24.21	24.47	8.42	70.87
Niger	-4.31	-5.13	-3.49	0.42	-21.62	24.00	8.22	67.56
Russia	-5.43	-5.73	-5.14	0.15	-24.71	23.85	6.71	44.99
Somalia	-7.28	-8.32	-6.25	0.52	-24.21	20.27	7.54	56.87

Supplementary Table 6: Computed statistics based on the multi-model ensemble mean of the predicted gird-cell level relative change in soil salinity (EC_e) in the long-term future (2071 - 2100) relative to the reference period (1961 - 1990) for the 30 countries with the highest number of dryland grid-cells in our analysis under RCP 4.5 greenhouse gas concentration trajectory.

RCP 4.5 long-term future (%)								
**Country**	**Mean**	**Meanci-low**	**Meanci-up**	**Sem**	**Min**	**Max**	**STDEV**	**Variance**
Namibia	12.36	11.36	13.36	0.51	-9.85	33.00	8.14	66.32
South Africa	9.50	8.32	10.68	0.60	-13.59	32.63	10.17	103.47
Brazil	6.55	5.14	7.96	0.72	-31.68	33.33	13.05	170.34
Mexico	5.68	4.70	6.67	0.50	-25.42	32.06	10.91	119.02
Mauritania	4.08	2.60	5.56	0.75	-21.73	32.97	13.15	172.81
Mali	3.04	1.71	4.38	0.68	-29.36	33.42	13.01	169.31
Botswana	2.77	1.11	4.42	0.84	-18.75	33.07	11.86	140.73
United States	2.73	2.14	3.33	0.30	-34.46	33.47	11.97	143.35
Niger	2.66	1.35	3.97	0.66	-26.86	33.31	12.20	148.89
Libya	2.54	1.62	3.46	0.47	-23.31	33.43	10.99	120.83
Chad	2.54	1.13	3.94	0.72	-31.16	33.43	13.80	190.35
Australia	2.26	1.93	2.59	0.17	-25.25	33.41	8.30	68.91
Argentina	2.01	1.27	2.74	0.37	-22.38	30.27	9.23	85.20
Pakistan	0.94	0.09	1.79	0.43	-28.16	29.71	7.16	51.29
China	0.34	-0.23	0.90	0.29	-34.53	33.48	11.37	129.19
Ethiopia	0.21	-0.92	1.34	0.57	-25.69	27.68	8.48	71.89
Bolivia	-0.24	-1.46	0.97	0.62	-21.33	26.61	8.96	80.26
Turkey	-0.24	-1.77	1.28	0.77	-23.34	31.18	12.01	144.31
Afghanistan	-0.33	-1.14	0.47	0.41	-12.54	27.95	6.27	39.29
Iran	-0.53	-1.17	0.11	0.33	-21.01	27.60	7.92	62.72
Somalia	-0.54	-2.09	1.00	0.78	-32.74	30.66	11.19	125.12
India	-0.76	-1.60	0.08	0.43	-31.17	33.07	10.69	114.37
Sudan	-1.81	-2.65	-0.97	0.43	-29.66	33.03	10.75	115.46
Canada	-2.87	-3.61	-2.13	0.38	-32.82	31.59	11.04	121.85
Algeria	-3.29	-4.17	-2.41	0.45	-32.13	33.55	12.02	144.42
Kazakhstan	-4.59	-5.16	-4.02	0.29	-34.60	31.66	10.28	105.77
Egypt	-5.18	-6.14	-4.21	0.49	-30.94	33.02	9.37	87.82
Russia	-6.25	-6.79	-5.70	0.28	-34.46	33.59	12.53	157.03
Mongolia	-7.46	-8.54	-6.39	0.55	-33.54	27.81	11.67	136.13
Saudi Arabia	-9.42	-10.02	-8.83	0.30	-32.65	33.41	7.86	61.75

Supplementary Table 7: Computed statistics based on the multi-model ensemble mean of the predicted gird-cell level relative change in soil salinity (EC_e) in the long-term future (2071 - 2100) relative to the reference period (1961 - 1990) for the 30 countries with the highest number of dryland grid-cells in our analysis under RCP 8.5 greenhouse gas concentration trajectory.

	RCP 8.5 long-term future (%)							
**Country**	**Mean**	**Meanci-low**	**Meanci-up**	**Sem**	**Min**	**Max**	**STDEV**	**Variance**
Brazil	15.10	13.25	16.95	0.94	-27.92	39.92	15.09	227.64
Namibia	13.57	12.10	15.04	0.75	-8.74	39.86	11.80	139.24
South Africa	11.20	9.41	13.00	0.91	-21.17	39.96	14.48	209.64
Mexico	6.38	4.96	7.80	0.72	-40.77	39.84	15.17	230.00
Botswana	6.06	4.28	7.83	0.90	-19.94	39.66	12.52	156.81
Mauritania	5.90	3.99	7.81	0.97	-22.07	39.73	16.50	272.32
Argentina	4.76	3.83	5.69	0.47	-26.49	39.38	11.68	136.47
Bolivia	4.34	2.53	6.15	0.92	-21.73	39.11	13.31	177.22
United States	3.70	3.02	4.39	0.35	-34.41	39.72	13.66	186.59
Australia	3.31	2.88	3.73	0.22	-21.55	39.96	10.57	111.67
Libya	2.80	1.38	4.22	0.72	-21.55	39.95	15.91	253.12
Somalia	2.77	0.98	4.56	0.91	-30.59	29.54	12.92	167.00
Pakistan	2.69	1.83	3.54	0.43	-16.62	28.51	7.23	52.24
Chad	2.38	0.72	4.03	0.84	-31.89	40.02	16.31	265.92
Niger	2.19	0.88	3.51	0.67	-25.42	37.94	12.50	156.19
Ethiopia	1.66	0.08	3.24	0.80	-29.61	38.14	11.76	138.39
Mali	1.05	-0.41	2.52	0.75	-27.74	39.35	14.61	213.55
Turkey	-0.39	-2.55	1.77	1.10	-29.74	39.12	16.72	279.59
China	-0.80	-1.60	-0.01	0.41	-41.24	40.02	15.51	240.69
Sudan	-1.28	-2.42	-0.14	0.58	-33.08	39.15	14.51	210.49
Canada	-2.09	-2.91	-1.28	0.41	-26.59	35.76	12.10	146.53
Afghanistan	-2.12	-2.85	-1.39	0.37	-14.46	19.16	5.66	32.01
India	-2.20	-2.89	-1.50	0.35	-23.77	36.78	9.07	82.33
Kazakhstan	-5.01	-5.52	-4.49	0.26	-33.99	39.59	9.25	85.47
Russia	-6.13	-6.61	-5.65	0.25	-32.31	40.05	11.04	121.79
Egypt	-6.47	-7.65	-5.29	0.60	-21.77	34.51	11.51	132.47
Iran	-6.99	-7.70	-6.28	0.36	-33.32	32.16	8.82	77.71
Algeria	-7.33	-8.35	-6.31	0.52	-29.26	39.74	13.87	192.47
Mongolia	-8.78	-10.22	-7.34	0.73	-37.41	37.14	15.58	242.64
Saudi Arabia	-12.96	-13.45	-12.46	0.25	-34.86	18.45	6.56	43.08

Supplementary Table 8: Computed statistics based on the multi-model ensemble mean of the predicted gird-cell level relative change in soil salinity (EC_e) in the long-term future (2071 - 2100) relative to the reference period (1961 - 1990) for the 30 countries with the highest number of dryland grid-cells in our analysis under SSP 2-4.5 greenhouse gas concentration trajectory.

	SSP 2-4.5 long-term future (%)							
**Country**	**Mean**	**Meanci-low**	**Meanci-up**	**Sem**	**Min**	**Max**	**STDEV**	**Variance**
South Africa	10.49	9.51	11.47	0.50	-12.10	29.16	8.48	71.88
Botswana	9.73	8.84	10.62	0.45	-4.79	25.48	6.40	40.93
Australia	9.31	8.97	9.65	0.17	-12.48	29.42	8.22	67.60
Namibia	8.57	7.70	9.44	0.44	-7.92	29.00	7.31	53.46
Brazil	7.78	6.73	8.83	0.53	-20.67	29.35	10.05	101.01
Mongolia	7.71	6.07	9.36	0.83	-13.39	28.69	11.42	130.38
Mexico	5.40	4.57	6.23	0.42	-17.17	29.15	9.27	86.01
Mauritania	4.05	3.38	4.72	0.34	-18.78	25.51	6.53	42.70
Mali	2.41	1.82	3.00	0.30	-15.53	22.84	6.12	37.44
Bolivia	1.57	0.50	2.64	0.54	-17.18	24.38	7.97	63.49
Algeria	1.04	0.58	1.50	0.24	-13.30	28.53	6.74	45.44
Sudan	1.01	0.37	1.65	0.33	-19.91	29.35	8.19	67.11
Libya	0.89	0.34	1.45	0.28	-26.50	29.13	6.67	44.46
Afghanistan	0.60	-0.20	1.40	0.41	-14.23	22.22	6.20	38.47
Egypt	0.35	-0.15	0.86	0.26	-12.05	15.36	4.89	23.93
China	-0.15	-0.67	0.37	0.26	-26.51	29.37	9.82	96.47
Argentina	-1.03	-1.58	-0.48	0.28	-28.14	27.12	6.95	48.32
United States	-1.32	-1.85	-0.80	0.27	-28.23	29.30	10.78	116.18
Saudi Arabia	-1.80	-2.30	-1.31	0.25	-19.13	16.06	6.62	43.79
Chad	-1.84	-2.60	-1.09	0.38	-26.42	23.57	7.73	59.71
Pakistan	-1.93	-2.73	-1.12	0.41	-16.99	25.26	6.82	46.48
Iran	-2.97	-3.47	-2.48	0.25	-18.90	18.71	6.11	37.35
Turkey	-3.09	-4.21	-1.98	0.57	-22.71	27.78	8.90	79.15
Niger	-3.23	-3.98	-2.48	0.38	-22.85	26.55	7.69	59.06
India	-4.23	-4.93	-3.52	0.36	-28.68	25.30	9.14	83.59
Kazakhstan	-4.30	-4.78	-3.81	0.25	-25.59	29.33	8.72	76.04
Canada	-4.76	-5.19	-4.33	0.22	-27.63	20.49	6.45	41.54
Ethiopia	-4.92	-6.20	-3.64	0.65	-26.72	26.95	9.58	91.73
Russia	-6.95	-7.30	-6.60	0.18	-28.39	28.68	7.81	60.96
Somalia	-9.91	-11.19	-8.63	0.65	-28.19	24.05	9.28	86.16

Supplementary Table 9: Computed statistics based on the multi-model ensemble mean of the predicted gird-cell level relative change in soil salinity (EC_e) in the long-term future (2071 - 2100) relative to the reference period (1961 - 1990) for the 30 countries with the highest number of dryland grid-cells in our analysis under SSP 5-8.5 greenhouse gas concentration trajectory.

	SSP 5-8.5 long-term future (%)							
**Country**	**Mean**	**Meanci-low**	**Meanci-up**	**Sem**	**Min**	**Max**	**STDEV**	**Variance**
Botswana	24.94	22.71	27.16	1.12	-5.89	44.10	13.10	171.74
South Africa	21.35	19.84	22.85	0.76	-15.09	43.32	12.51	156.41
Namibia	17.69	16.14	19.24	0.79	-7.07	43.52	12.54	157.13
Brazil	16.21	14.77	17.66	0.73	-16.80	44.09	13.07	170.80
Australia	14.60	14.17	15.03	0.22	-41.51	44.12	10.39	107.91
Mexico	12.66	11.38	13.94	0.65	-19.32	43.38	13.87	192.43
Mongolia	6.18	3.67	8.69	1.27	-34.40	44.21	20.04	401.77
Mali	5.76	4.78	6.73	0.50	-14.98	42.29	10.10	102.05
Mauritania	4.12	3.25	4.99	0.44	-14.54	31.63	8.45	71.46
Afghanistan	3.15	2.17	4.14	0.50	-17.56	25.87	7.64	58.37
Libya	2.87	2.20	3.55	0.35	-19.15	41.13	8.14	66.26
Bolivia	2.37	0.82	3.92	0.79	-19.19	42.96	11.55	133.39
Argentina	1.57	0.82	2.32	0.38	-21.11	43.57	9.40	88.40
Egypt	0.44	-0.14	1.01	0.29	-13.14	34.62	5.62	31.57
Sudan	0.43	-0.47	1.32	0.46	-24.87	43.55	11.46	131.34
Turkey	-0.04	-1.82	1.74	0.90	-26.92	42.28	14.18	201.19
United States	-0.11	-0.89	0.67	0.40	-39.52	42.90	15.91	253.14
China	-0.72	-1.39	-0.06	0.34	-38.33	43.81	13.09	171.32
Algeria	-1.14	-1.84	-0.44	0.36	-22.39	42.55	10.10	102.10
Pakistan	-1.15	-2.35	0.05	0.61	-20.74	37.28	10.14	102.91
India	-1.92	-2.76	-1.07	0.43	-27.94	42.21	10.96	120.06
Saudi Arabia	-2.64	-3.33	-1.96	0.35	-24.21	21.90	9.13	83.42
Iran	-2.79	-3.49	-2.09	0.35	-23.79	40.07	8.60	73.92
Ethiopia	-2.95	-4.88	-1.02	0.98	-30.30	41.58	14.40	207.24
Chad	-4.89	-5.91	-3.87	0.52	-28.99	39.68	10.48	109.82
Niger	-5.68	-6.88	-4.49	0.61	-40.41	41.24	12.13	147.14
Kazakhstan	-7.00	-7.61	-6.39	0.31	-32.99	43.51	11.05	121.99
Canada	-7.10	-7.60	-6.59	0.26	-31.75	24.19	7.54	56.80
Russia	-7.95	-8.44	-7.46	0.25	-40.19	43.63	11.21	125.58
Somalia	-12.03	-13.84	-10.22	0.92	-37.56	36.36	13.30	176.98

Supplementary Table 10: Countries with the highest number of dryland grid-cells and the estimates of their total dryland area used in the present study.

Country	Number of grid-cells	Total dryland area (km^2)
Australia	2,421	6,717,230.56
United States	1,647	3,907,880.47
China	1,595	3,697,849.00
Russia	2,056	3,660,984.24
Kazakhstan	1,271	2,619,580.23
Algeria	825	2,243,524.51
Saudi Arabia	684	1,921,997.39
Sudan	633	1,870,833.54
India	656	1,859,113.43
Libya	582	1,597,000.93
Canada	862	1,555,847.54
Iran	590	1,537,911.25
Argentina	620	1,490,518.78
Mexico	511	1,414,650.16
Brazil	429	1,294,077.03
Mali	418	1,226,669.13
Chad	409	1,211,794.68
Niger	404	1,186,006.78
Mauritania	369	1,065,585.74
Egypt	366	1,009,647.31
Mongolia	463	999,368.89
South Africa	317	853,035.45
Namibia	281	801,629.98
Pakistan	278	749,601.24
Ethiopia	222	675,178.49
Somalia	210	641,504.11
Bolivia	217	634,507.78
Turkey	253	608,965.38
Afghanistan	234	601,733.75
Botswana	206	588,102.91

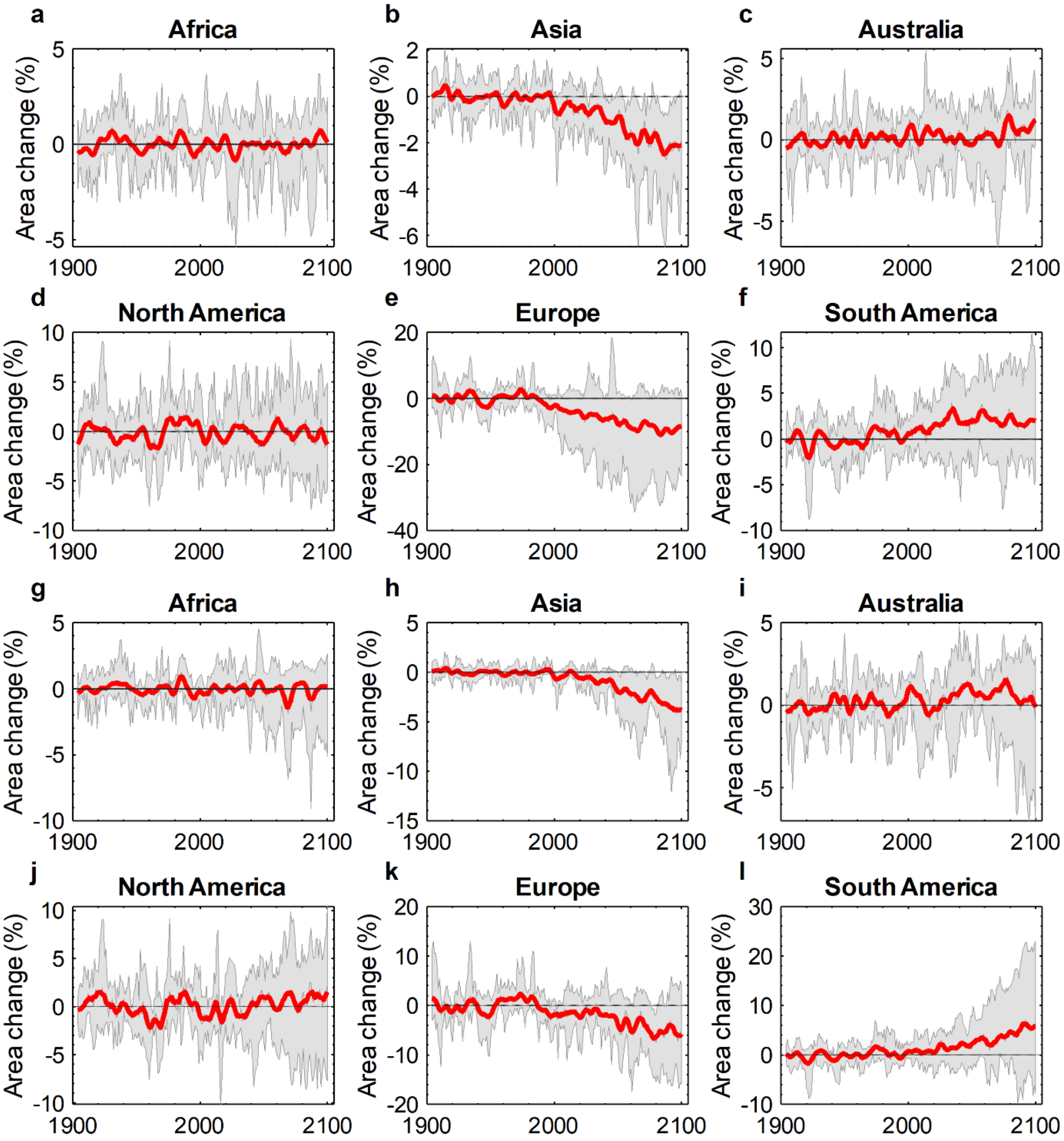

Supplementary Fig. 4: Continental-level predicted annual change in the total area of soils with an $EC_e \geq 2$ dS m^{-1} relative to the 20th century average (1904 - 1999) for the models obtained from CMIP5 data project. **a** to **f**: Relative change under RCP 4.5 greenhouse gas concentration trajectory. **g** to **l**: Relative change under RCP 8.5 greenhouse gas concentration trajectory. Shaded areas show the minimum and maximum range of the relative changes predicted by multi-model ensemble members. Red lines show the low-pass filtered (5-year running window) of the multi-model ensemble mean of the predicted variations; since all spatio-temporal predictors are five year moving averages, 1904 is the beginning of the period.

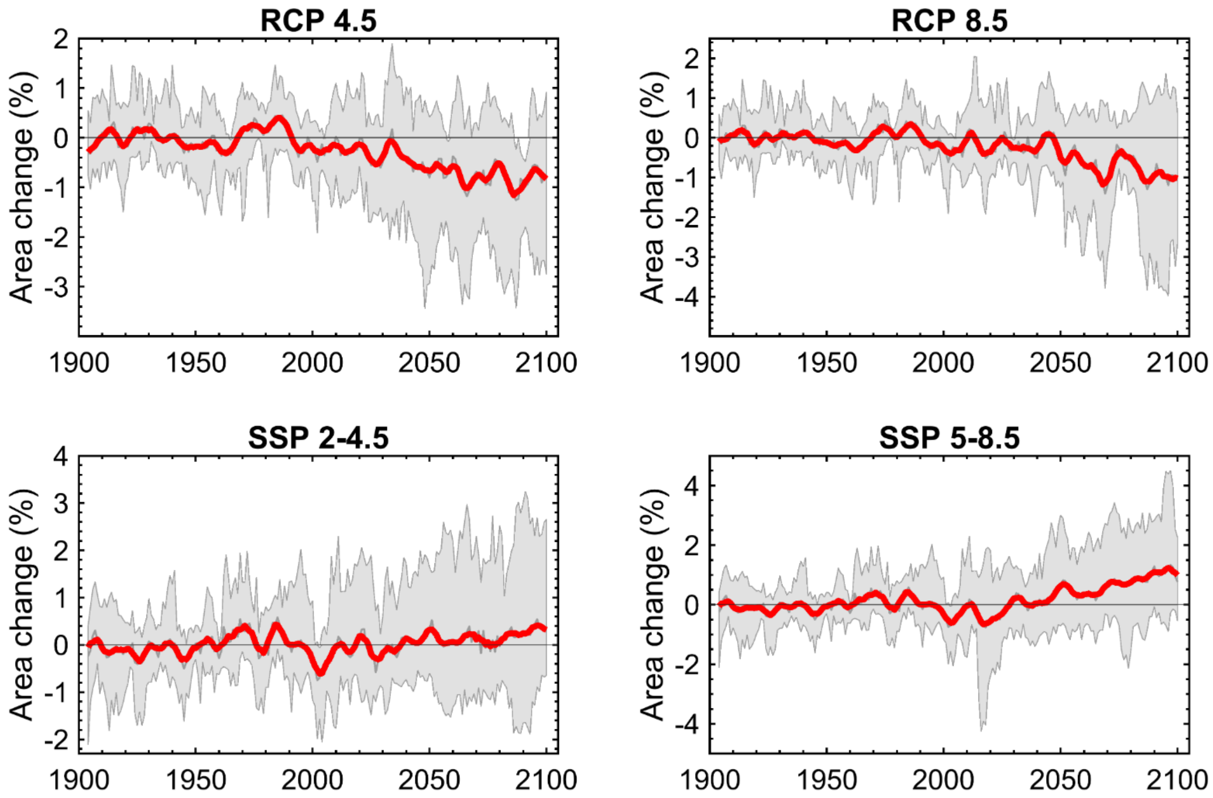

Supplementary Fig. 5: Predicted annual change in the total area of soils with an $EC_e \geq 2$ dS m^{-1} relative to the average of 1904 - 1999 period under different greenhouse gas concentration trajectories on the global scale. Shaded areas show the minimum and maximum range of the relative changes predicted by multi-model ensemble members. Red lines show the low-pass filtered (5-year running window) of the multi-model ensemble mean of the predicted variations; since all spatio-temporal predictors are five year moving averages, 1904 is the beginning of the period.

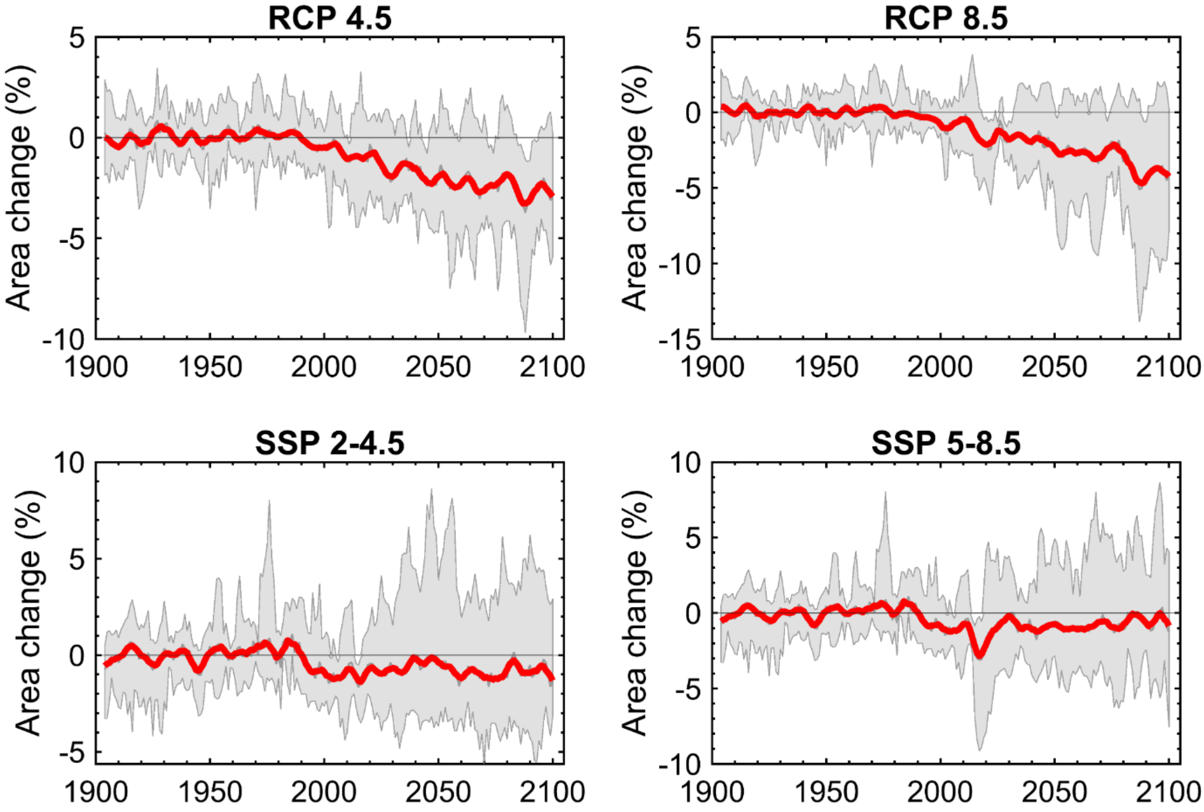

Supplementary Fig. 6: Predicted annual change in the total area of soils with an $EC_e \geq 4$ dS m^{-1} relative to the average of 1904 - 1999 period under different greenhouse gas concentration trajectories on the global scale. Shaded areas show the minimum and maximum range of the relative changes predicted by multi-model ensemble members. Red lines show the low-pass filtered (five-year running window) of the multi-model ensemble mean of the predicted variations; since all spatio-temporal predictors are five-year moving averages, 1904 is the beginning of the period.

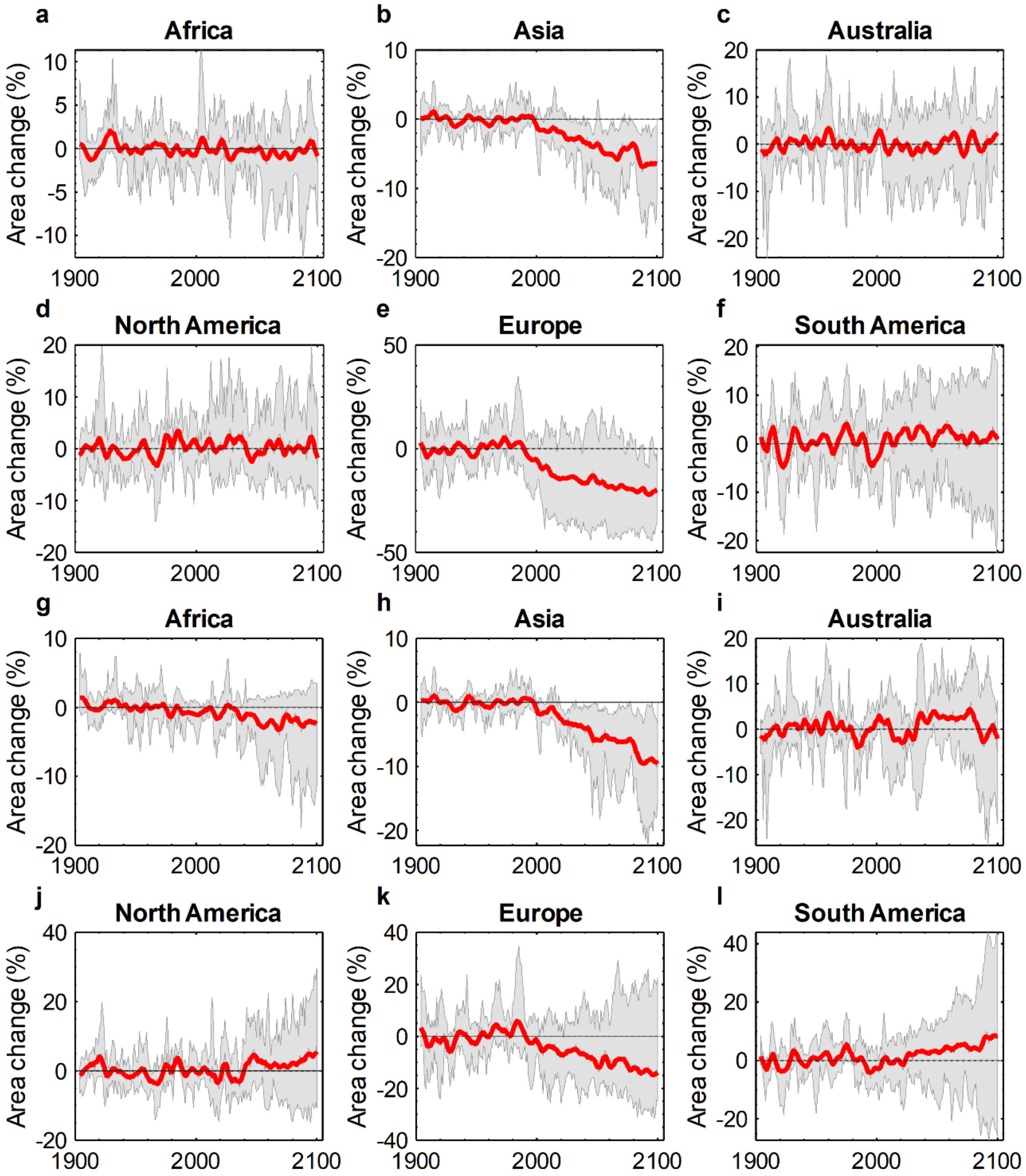

Supplementary Fig. 7: Continental-level predicted annual change in the total area of soils with an $EC_e \geq 4$ dS m^{-1} relative to the 20th century average (1904 - 1999) for the models obtained from the CMIP5 data. a to **f**: Relative change under RCP 4.5 greenhouse gas concentration trajectory. **g** to **l**: Relative change under RCP 8.5 greenhouse gas concentration trajectory. Shaded areas show the minimum and maximum range of the relative changes predicted by multi-model ensemble members. Red lines show the low-pass filtered (five-year running window) of the multi-model ensemble mean of the predicted variations; since all spatio-temporal predictors are five-year moving averages, 1904 is the beginning of the period.

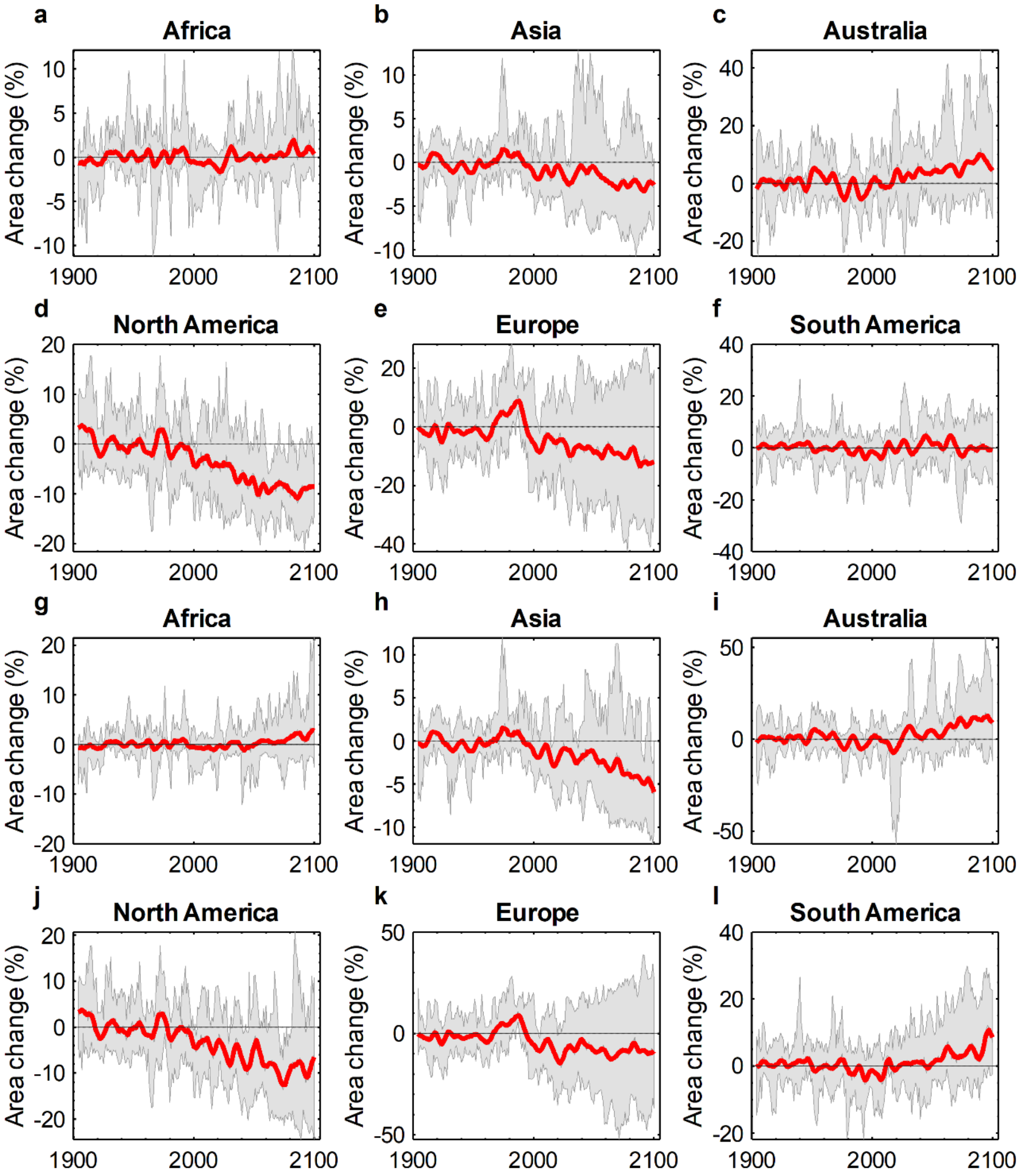

Supplementary Fig. 8: Continental-level predicted annual change in the total area of soils with an $EC_e \geq 4$ dS m^{-1} relative to the 20th century average (1904 - 1999) for the models obtained from CMIP6 data project. **a** to **f**: Relative change under SSP 2-4.5 greenhouse gas concentration trajectory. **g** to **l**: Relative change under SSP 5-8.5 greenhouse gas concentration trajectory. Shaded areas show the minimum and maximum range of the relative changes predicted by multi-model ensemble members. Red lines show the low-pass filtered (five-year running window) of the multi-model ensemble mean of the predicted variations; since all spatio-temporal predictors are five-year moving averages, 1904 is the beginning of the period.

Supplementary Table 11: Country-level predicted change in the total area of soils with an $EC_e \geq 2$ dS m^{-1} at the long-term future (2071 - 2100), compared to the 20th century average (1904 - 1999) under different greenhouse gas concentration trajectories.

Country	Scenarios							
	RCP 4.5 mid-term future (%)	RCP 4.5 long-term future (%)	RCP 8.5 mid-term future (%)	RCP 8.5 long-term future (%)	SSP 2-4.5 mid-term future (%)	SSP 2-4.5 long-term future (%)	SSP 5-8.5 mid-term future (%)	SSP 5-8.5 long-term future (%)
Afghanistan	-1.69	-2.55	-3.05	-4.29	-0.60	-0.33	-0.45	-0.28
Algeria	0.38	0.98	0.57	-0.03	0.36	0.48	0.55	0.68
Argentina	0.02	-1.68	-0.12	-0.36	-0.95	-1.03	-0.53	-0.11
Australia	0.02	0.70	0.79	0.60	1.59	2.40	1.36	3.38
Bolivia	-1.33	-2.79	-0.03	-1.52	0.47	0.30	-0.06	-0.54
Botswana	1.03	3.63	3.74	4.82	5.59	5.11	5.13	7.52
Brazil	12.19	14.32	8.57	29.50	18.69	23.92	24.81	43.08
Canada	-4.22	-5.86	-3.19	-5.85	-5.01	-6.96	-5.38	-9.98
Chad	-2.47	-2.23	-2.02	-3.20	1.19	1.17	0.50	0.81
China	-1.00	-2.18	-1.65	-2.37	0.73	1.03	0.88	1.97
Egypt	-2.15	-2.25	-2.18	-5.74	0.21	0.23	0.15	0.21
Ethiopia	0.39	1.31	-0.08	3.83	-3.11	-6.23	-3.89	-4.99
India	-1.30	-1.60	-1.63	-0.92	-3.20	-3.24	-2.48	-2.94
Iran	0.20	0.49	-0.49	-1.88	-0.64	-1.45	-0.98	-1.47
Kazakhstan	-1.02	-2.44	-0.27	-2.58	-0.14	-1.09	-0.15	-2.52
Libya	0.26	-0.07	-1.34	1.13	0.55	0.69	0.58	0.47
Mali	0.17	0.37	-0.68	-1.97	-0.76	-0.23	-0.77	0.33
Mauritania	1.43	1.88	2.47	4.85	-0.02	0.61	0.55	0.60
Mexico	4.32	6.54	0.29	12.57	0.69	6.75	5.13	14.51
Mongolia	-5.71	-9.22	-10.49	-13.30	4.59	7.29	5.76	8.05
Namibia	2.30	3.63	2.56	4.40	1.25	1.80	1.75	2.72
Niger	1.55	1.14	1.34	0.28	0.09	0.34	0.89	0.53
Pakistan	0.03	0.05	0.09	0.29	0.26	0.27	0.30	0.33
Russia	-4.45	-7.93	-2.50	-7.82	-1.71	-2.88	-2.01	-4.16
Saudi Arabia	-0.46	-1.48	-0.68	-2.63	0.28	0.20	0.04	0.56
Somalia	-1.75	-1.90	-1.82	0.88	-3.42	-6.22	-4.19	-8.53
South Africa	1.02	3.88	2.92	5.65	1.30	3.45	2.94	7.18
Sudan	-1.51	-2.15	-1.92	-4.16	-0.25	0.43	0.25	1.22
Turkey	-2.36	-1.41	-5.21	-5.35	-0.35	-2.83	-1.03	-1.00
United States	1.22	1.54	2.12	2.30	-3.59	-3.10	-2.57	-2.63

Supplementary Table 12: Continental-level predicted change in the total area of soils with an $EC_e \geq 4$ dS m^{-1} in the mid and long-term futures relative to the average of the 1904 - 1999 period under different greenhouse gas concentration trajectories.

Scenarios	Continent					
	Africa	Asia	Australia	North America	Europe	South America
RCP 4.5; mid-term (%)	-0.37	-3.73	-0.50	0.04	-15.72	2.27
RCP 4.5; long-term (%)	-0.32	-5.35	0.26	0.00	-20.07	1.03
RCP 8.5; mid-term (%)	-1.60	-4.80	2.38	1.21	-8.26	3.26
RCP 8.5; long-term (%)	-1.99	-7.99	0.65	3.04	-12.77	5.97
SSP 2-4.5; mid-term (%)	0.08	-1.17	3.76	-7.50	-8.14	1.00
SSP 2-4.5; long-term (%)	0.76	-2.56	6.78	-9.10	-10.79	-0.75
SSP 5-8.5; mid-term (%)	0.07	-1.89	2.94	-6.34	-6.96	1.09
SSP 5-8.5; long-term (%)	1.60	-4.26	10.40	-9.54	-8.25	5.19

Supplementary Table 13: Country-level predicted change in the total area of soils with an $EC_e \geq 4$ dS m^{-1} at the long-term future (2071 - 2100), compared to the 20th century average (1904 - 1999) under different greenhouse gas concentration trajectories.

Country	Scenarios							
	RCP 4.5 mid-term future (%)	RCP 4.5 long-term future (%)	RCP 8.5 mid-term future (%)	RCP 8.5 long-term future (%)	SSP 2-4.5 mid-term future (%)	SSP 2-4.5 long-term future (%)	SSP 5-8.5 mid-term future (%)	SSP 5-8.5 long-term future (%)
Afghanistan	-3.20	-4.57	-6.90	-10.31	-1.23	0.71	0.07	3.36
Algeria	-4.30	-3.74	-5.58	-12.48	-0.62	-1.90	-3.67	-2.91
Argentina	3.32	-0.24	3.06	2.70	-3.20	-7.19	-3.93	-4.92
Australia	-0.50	0.26	2.38	0.65	3.76	6.78	2.94	10.40
Bolivia	-2.78	-3.61	-0.96	-3.77	-3.65	-6.55	-5.53	-9.02
Botswana	-1.50	-0.34	2.93	4.28	14.37	11.59	15.79	25.44
Brazil	2.10	1.45	4.48	27.61	14.73	20.31	19.85	62.34
Canada	-3.82	-4.44	-7.06	-7.68	-9.45	-14.06	-10.30	-19.17
Chad	1.18	0.86	-0.84	3.28	-7.50	-4.54	-8.43	-9.06
China	-0.10	-0.59	-2.73	0.28	-1.32	-4.08	-1.93	-9.77
Egypt	-4.85	-7.45	-5.74	-11.77	0.13	-0.44	0.18	0.03
Ethiopia	-8.68	-9.84	-9.24	8.80	-7.51	-10.80	-9.37	-12.79
India	-4.81	-5.91	-4.98	-11.19	-5.97	-7.23	-5.30	-6.08
Iran	-3.54	-3.66	-6.96	-10.73	-2.12	-2.88	-2.16	-2.47
Kazakhstan	-7.01	-9.63	-6.27	-9.49	-3.49	-7.01	-4.09	-10.21
Libya	0.61	0.23	-0.79	1.18	-1.73	-0.91	-1.57	-0.97
Mali	-1.30	1.75	1.20	-3.33	5.96	8.94	11.28	22.00
Mauritania	2.13	3.12	4.61	7.55	5.30	7.30	6.87	1.48
Mexico	8.31	12.51	7.27	24.73	8.53	18.28	16.02	38.15
Mongolia	-14.66	-16.69	-16.47	-18.85	5.80	8.07	4.72	3.64
Namibia	10.63	14.75	8.44	15.37	4.85	8.54	6.29	14.99
Niger	7.52	10.29	9.22	10.63	-5.51	-3.71	-4.55	-4.70
Pakistan	1.32	0.28	-0.06	2.56	-1.25	-0.69	-0.83	-1.08
Russia	-15.57	-21.50	-13.97	-23.93	-11.35	-14.64	-11.05	-20.87
Saudi Arabia	-3.22	-5.81	-4.10	-10.96	4.48	3.13	0.92	3.79
Somalia	-3.81	0.64	-0.98	10.04	-6.69	-16.02	-9.46	-20.77
South Africa	8.88	18.62	10.71	24.53	6.73	14.10	10.08	24.38
Sudan	-4.46	-5.77	-6.13	-8.18	0.01	1.80	-1.63	0.85
Turkey	-10.34	-7.68	-17.97	0.21	-3.98	-10.04	-6.91	-8.31
United States	1.09	0.73	5.03	6.38	-9.74	-11.17	-8.39	-12.67

Supplementary Table 14: The optimal values of hyperparameters and goodness-of-fit quantified by accuracy metrics for the final 16 best fitted models.

Model name	Number of learning cycles	Learn rate	Minimum leaf size	Maximum number of splits	Number of variables to sample	*MSE*[a]	*RMSE*[b]	*MAE*[c]	*NSE*[d]	R^2	Minimum Objective function	Ensemble aggregation method
CMIP5 models												
GISS-E2-H	210	0.090	14	487	2	13.30	3.65	1.60	0.72	0.72	2.659	LSBoost
GISS-E2-R	79	0.101	20	21,996	3	13.47	3.67	1.57	0.72	0.72	2.712	LSBoost
MIROC5-ensemble	69	0.137	8	35,913	2	13.11	3.62	1.51	0.72	0.72	2.737	LSBoost
MIROC-ESM-CHEM	337	0.039	6	1,824	1	12.68	3.56	1.45	0.73	0.73	2.643	LSBoost
MIROC-ESM	38	0.146	16	640	6	12.61	3.55	1.41	0.73	0.73	2.766	LSBoost
MRI-CGCM3	73	0.107	9	8,266	3	12.83	3.58	1.47	0.73	0.73	2.652	LSBoost
MRI-ESM1	43	0.135	3	2,290	7	13.39	3.66	1.54	0.72	0.72	2.736	LSBoost
NorESM1-M	66	0.110	3	3,375	3	13.05	3.61	1.45	0.73	0.73	2.729	LSBoost
CMIP6 models												
CESM2-WACCM-ensemble	210	0.090	14	487	2	13.30	3.65	1.60	0.72	0.72	2.659	LSBoost
CNRM-ESM2-1	79	0.101	20	21,996	3	13.47	3.67	1.57	0.72	0.72	2.712	LSBoost
GFDL-ESM4	69	0.137	8	35,913	2	13.11	3.62	1.51	0.72	0.72	2.737	LSBoost
INM-CM4-8-	337	0.039	6	1,824	1	12.68	3.56	1.45	0.73	0.73	2.643	LSBoost
INM-CM5-0	38	0.146	16	640	6	12.61	3.55	1.41	0.73	0.73	2.766	LSBoost
MIROC-ES2L	73	0.107	9	8,266	3	12.83	3.58	1.47	0.73	0.73	2.652	LSBoost
MRI-ESM2-0	43	0.135	3	2,290	7	13.39	3.66	1.54	0.72	0.72	2.736	LSBoost
NorESM2-LM	66	0.110	3	3,375	3	13.05	3.61	1.45	0.73	0.73	2.729	LSBoost

[a] Mean Squared Error.
[b] Root Mean Squared Error.
[c] Mean Absolute Error.
[d] Nash–Sutcliffe Model Efficiency Coefficient.

Supplementary Table 15: Lower and upper limits of 95% confidence intervals of the mean for 10-fold cross-validation accuracy metrics calculated for trained models. We repeated the model trainings and hyperparameter tuning jobs for each of 16 datasets for 30 times and calculated confidence intervals of the mean based on 1,000 bootstrapped samples (with replacement) derived from the 30 validation metrics.

**Model name**	***MSE***		***RMSE***		***MAE***		***NSE***		R^2		**Minimum Objective function**	
	Lower	Upper	Lower	Upper	Lower	Upper	Lower	Upper	Lower	Upper	Lower	Upper
CMIP5 models												
GISS-E2-H	14.331	15.420	3.784	3.925	1.612	0.698	0.675	0.698	0.676	0.699	2.766	2.820
GISS-E2-R	14.331	15.420	3.784	3.925	1.612	0.698	0.675	0.698	0.676	0.699	2.766	2.820
MIROC5-ensemble	13.874	14.595	3.724	3.819	1.572	0.708	0.693	0.708	0.694	0.709	2.728	2.795
MIROC-ESM-CHEM	13.776	15.018	3.708	3.871	1.573	0.710	0.684	0.710	0.685	0.710	2.708	2.781
MIROC-ESM	13.300	14.194	3.647	3.766	1.518	0.720	0.701	0.720	0.702	0.720	2.667	2.755
MRI-CGCM3	13.968	15.233	3.735	3.900	1.583	0.706	0.679	0.706	0.681	0.707	2.719	2.792
MRI-ESM1	14.221	15.307	3.770	3.910	1.552	0.701	0.678	0.701	0.681	0.702	2.728	2.794
NorESM1-M	14.363	15.592	3.786	3.944	1.576	0.698	0.672	0.698	0.674	0.699	2.768	2.826
CMIP6 models												
CESM2-WACCM-ensemble	13.402	14.584	3.658	3.816	1.544	0.718	0.693	0.718	0.694	0.718	2.687	2.776
CNRM-ESM2-1	14.174	15.675	3.758	3.955	1.591	0.702	0.670	0.702	0.673	0.703	2.738	2.805
GFDL-ESM4	14.127	15.361	3.757	3.918	1.556	0.703	0.677	0.703	0.681	0.704	2.741	2.818
INM-CM4-8-	14.179	15.327	3.763	3.912	1.574	0.701	0.677	0.701	0.679	0.703	2.738	2.817
INM-CM5-0	13.753	14.842	3.706	3.849	1.586	0.710	0.687	0.710	0.688	0.711	2.745	2.781
MIROC-ES2L	14.264	15.267	3.774	3.904	1.572	0.700	0.679	0.700	0.681	0.701	2.743	2.809
MRI-ESM2-0	14.300	15.263	3.778	3.905	1.590	0.699	0.679	0.699	0.680	0.700	2.735	2.802
NorESM2-LM	14.262	15.147	3.775	3.891	1.623	0.700	0.681	0.700	0.685	0.701	2.709	2.810
Average	14.020	15.122	3.742	3.886	1.575	0.705	0.682	0.705	0.684	0.706	2.728	2.799

Supplementary Table 16: Coefficients of determination (R^2) between measured and predicted values of soil salinity (EC_e) by 16 best fitted models at different soil depth intervals.

**Model name**	**Depth below the surface (cm)**					
	**0 - 20**	**20 - 40**	**40 - 60**	**60 - 80**	**80 - 100**	**100 - 200**
CMIP5 models						
GISS-E2-H	0.62	0.73	0.76	0.76	0.78	0.71
GISS-E2-R	0.62	0.73	0.76	0.76	0.78	0.71
MIROC5-ensemble	0.64	0.73	0.76	0.78	0.80	0.72
MIROC-ESM-CHEM	0.65	0.74	0.79	0.77	0.81	0.73
MIROC-ESM	0.64	0.74	0.80	0.79	0.82	0.73
MRI-CGCM3	0.64	0.74	0.78	0.77	0.79	0.73
MRI-ESM1	0.63	0.72	0.77	0.77	0.80	0.72
NorESM1-M	0.62	0.72	0.75	0.77	0.80	0.73
CMIP6 models						
CESM2-WACCM-ensemble	0.64	0.73	0.77	0.78	0.80	0.73
CNRM-ESM2-1	0.66	0.74	0.78	0.78	0.80	0.73
GFDL-ESM4	0.63	0.73	0.79	0.77	0.79	0.73
INM-CM4-8-	0.64	0.72	0.77	0.78	0.81	0.74
INM-CM5-0	0.65	0.74	0.77	0.76	0.79	0.72
MIROC-ES2L	0.62	0.72	0.76	0.77	0.77	0.71
MRI-ESM2-0	0.64	0.72	0.78	0.78	0.80	0.73
NorESM2-LM	0.63	0.74	0.78	0.78	0.79	0.72
Average	0.64	0.73	0.77	0.77	0.80	0.73

Computer codes

This section makes the scripts and codes, required to regenerate the results, available to readers. Please note ArcGIS Desktop 10.x license is needed to run ArcPy module. Also, MATLAB Parallel Computing plus Statistics and Machine Learning toolboxes are required for running the MATLAB codes provided here.

Purely spatial predictors were directly pre-processed (including projections and resampling) in ArcGIS for Desktop environment (herein we refer to its central application: ArcMap). Soil texture raster datasets of clay content at different depths were averaged using ArcMap "raster calculator" tool. We extracted the values of predictors at the training input profiles locations by ArcMap "Extract Multi Values to Points" tool and saved the results as a table in .text format. The netCDF files of spatio-temporal predictors were processed by Climate Data Operators software and converted to multi-band rasters by ArcMap "Make NetCDF Raster Layer" tool. We extracted the values of these predictors at the locations of input profiles data too and saved them as tables in .text format. At each location there were 201 values, representing the years between 1900 and 2100. These data were finally merged, five-year moving averages of the spatio-temporal predictors were calculated and we attributed the moving averages of values of the spatio-temporal predictors at the locations to the input profiles data based on the year of acquisition of the sample profile using the following code:

```
clc;
clear;

%% This code merges the purely spatial and spatio-temporal predictors for each of the 16 input
datasets and prepare them for model training

% Importing the static predictors input data table
Soil_pro = readtable('D:\Projection_ML\Training_data_spatial\...
Training_data_static_predictors.txt','FileType','text','Delimiter','comma');

Model_name = {'Model_name_1'...
    'Model_name_2'...
    'Model_name_n'};


for ii = 1:length(Model_name) % Repeating the process for all 16 models

    % Importing input spatio-temporal data tables computed based on the
    % output of each of the 29 GCMs
    dryss_table = readtable(strcat('D:\Projection_ML\Training_data_spatio-...
    temporal\',Model_name{ii},...
    '\dryss_',Model_name{ii},'.txt'),'FileType','text','Delimiter','comma');
    wetss_table = readtable(strcat('D:\Projection_ML\Training_data_spatio-...
    temporal\',Model_name{ii},'\wetss_',Model_name{ii},'.txt'), ...
    'FileType','text','Delimiter','comma');
    evspsbl_table = readtable(strcat('D:\Projection_ML\Training_data_spatio-  ...
    temporal\',Model_name{ii},'\evspsbl_',Model_name{ii},'.txt'), ...
   'FileType','text','Delimiter','comma');
    pr_mean_table = readtable(strcat('D:\Projection_ML\Training_data_spatio-...
    temporal\',Model_name{ii},'\pr_mean_',Model_name{ii},'.txt'), ...
    'FileType','text','Delimiter','comma');
    pr_fre_table = readtable(strcat('D:\Projection_ML\Training_data_spatio- ...
    temporal\',Model_name{ii},'\pr_fre_',Model_name{ii},'.txt'), ...
    'FileType','text','Delimiter','comma');


    % Joining the tables
    T_dryss = ...
    join(Soil_pro,dryss_table,'LeftKeys','profile_id','RightKeys','Base_points_summarised');
    T_wetss = ...
    join(Soil_pro,wetss_table,'LeftKeys','profile_id','RightKeys','Base_points_summarised');
    T_evspsbl = ...
    join(Soil_pro,evspsbl_table,'LeftKeys','profile_id','RightKeys','Base_points_summarised');
    T_pr_mean = ...
    join(Soil_pro,pr_mean_table,'LeftKeys','profile_id','RightKeys','Base_points_summarised');
```

```
    T_pr_fre =  ...
    join(Soil_pro,pr_fre_table,'LeftKeys','profile_id','RightKeys','Base_points_summarised');

    % Calculation of the five-year moving averages
    Year_matrix = table2array(Soil_pro(:,7));
    dryss_matrix = table2array(T_dryss(:,22:222));
    dryss_matrix = movmean(dryss_matrix,[4 0],2,'omitnan','Endpoints','discard');
    wetss_matrix = table2array(T_wetss(:,22:222));
    wetss_matrix = movmean(wetss_matrix,[4 0],2,'omitnan','Endpoints','discard');
    evspsbl_matrix = table2array(T_evspsbl(:,22:222));
    evspsbl_matrix = movmean(evspsbl_matrix,[4 0],2,'omitnan','Endpoints','discard');
    pr_mean_matrix = table2array(T_pr_mean(:,22:222));
    pr_mean_matrix = movmean(pr_mean_matrix,[4 0],2,'omitnan','Endpoints','discard');
    pr_fre_matrix = table2array(T_pr_fre(:,22:222));
    pr_fre_matrix = movmean(pr_fre_matrix,[4 0],2,'omitnan','Endpoints','discard');

    dryss = zeros(size(Year_matrix,1),1);
    wetss = zeros(size(Year_matrix,1),1);
    evspsbl = zeros(size(Year_matrix,1),1);
    pr_mean = zeros(size(Year_matrix,1),1);
    pr_fre = zeros(size(Year_matrix,1),1);


    % Attributing spatio-temporal data to location of the points based on
    % the year of sampling the profile
    for i = 1:size(Year_matrix,1)
        dryss(i,1) = dryss_matrix(i,Year_matrix(i,1)-1903);
        wetss(i,1) = wetss_matrix(i,Year_matrix(i,1)-1903);
        evspsbl(i,1) = evspsbl_matrix(i,Year_matrix(i,1)-1903);
        pr_mean(i,1) = pr_mean_matrix(i,Year_matrix(i,1)-1903);
        pr_fre(i,1) = pr_fre_matrix(i,Year_matrix(i,1)-1903);
    end

    input_matrix = [Soil_pro.profile_id Soil_pro.profile_la Soil_pro.POINT_X Soil_pro.POINT_Y
...
    Soil_pro.Year...
        Soil_pro.upper_dept Soil_pro.lower_dept Soil_pro.WRB Soil_pro.Clay...
        Soil_pro.Elev Soil_pro.Slope Soil_pro.Field_capa Soil_pro.Wilt_point
        Soil_pro.Root_depth...
        dryss wetss evspsbl pr_fre pr_mean Soil_pro.elcosp_v_1];
    Table = array2table(input_matrix,'VariableNames',{'profile_id' 'profile_la' 'X' 'Y'
'Year'...
        'upper_dept' 'lower_dept' 'WRB' 'Clay'...
        'Elevation' 'Slope' 'Field_capa' 'Wilt_point' 'Root_depth' 'dryss'...
        'wetss' 'evspsbl' 'pr_fre' 'pr_mean' 'ECe'});

    % Saving output in a table format on disk
    writetable(Table,strcat('D:\Projection_ML\Training_input\',Model_name{ii},'.txt'));

end
```

16 input tables were generated for model training. These tables were later imported to MATAB to train an ensemble of regression tree learners using the following code:

```
clc;
clear;

%% Fitting an ensemble of regression trees for the 16 input datasets
% This script returns the tuned fitrensemble hyperparameters for 30 iterations using
fitrensemble % function in order to calculate the confidence intervals using bootstrapping
technique.
% This is regression using ensemble of trees (fitrensemble) on ECe as
% a target variable


Model_name = {'Model_name_1'...
    'Model_name_2'...
    'Model_name_n'};

for ii = 1:length(Model_name) % Repeating the process for all 16 models
    % Importing the original datasets prepared for training
    % Preprocessing the original dataset
    ECe = ...
```

```
    readtable(strcat('D:\Projection_ML\Training_input\',Model_name{ii},'.txt'),'FileType',...
        'text','Delimiter',',','PreserveVariableNames',true);

    table = standardizeMissing(ECe,-9999); % Replacing the missing values with -9999
    table(sum(ismissing(table),2) > 0,:) = [];% dropping the rows with missing values
    X = table(:,6:20);
    X.WRB = categorical(X.WRB);% Categorizing the categorical variables in the training set

    % Training and hyperparameter tuning job for 30 times
    % We used holdout method (25% held out) with the maximum 100 objective
    % function evaluations to optimize the ensemble hyperparameters
    % "ens" is the object of the final trained model
    for i = 1:30
        ens = fitrensemble(X,'ECe',...
            'OptimizeHyperparameters','all',...
            'HyperparameterOptimizationOptions',struct('Holdout',0.25,'UseParallel',true,...
            'MaxObjectiveEvaluations',100,'Repartition',true,'ShowPlots',false,'Verbose',1));
        % Acquiring and saving hyperparameter tuning job results on disk


save(strcat('D:\Projection_ML\Trained_ensemble\',Model_name{ii},'\ens_',num2str(i)),'ens');
    end
end
```

The training process on each of the 16 input datasets was repeated for 30 times. Using the following code, we cross-validated the models, calculated the accuracy metrics, and built their 95% confidence intervals of the mean:

```
clc;
clear;


%% Calculation of the confidence intervals of the mean for 10-fold cross-validation accuracy
% metrics
% Accuracy metrics including mean squared error (mse), mean absolute error (mae),
% NSE, and coefficient of determination (R^2) are computed

Model_name = {'Model_name_1'...
    'Model_name_2'...
    'Model_name_n'};

for ii = 1:length(Model_name) % Repeating the process for all 16 models

    ECe = ...
    readtable(strcat('D:\Projection_ML\Training_input\',Model_name{ii},'.txt'),'FileType',...
        'text','Delimiter',',','PreserveVariableNames',true);

    table = standardizeMissing(ECe,-9999);
    table(sum(ismissing(table),2) > 0,:) = [];

    % % Pre-allocating memory to variables with increasing size in each iteration
    Num_learning_cycles = zeros(30,1); Learn_rate = zeros(30,1); Min_leaf_size = zeros(30,1);
    Max_num_splits = zeros(30,1); Num_variables_to_sample = zeros(30,1);
    mse = zeros(30,1); mae = zeros(30,1);NSE = zeros(30,1); MinObjective = zeros(30,1);
    R2= zeros(30,1); Method = cell(30,1);

    % % Validation and Acquiring accuracy metrics
        ytrue = table.ECe;
    % This loop cross-validates the fitted models using 10-fold cross-validation
    for i = 1:30
        % Loading the saved model objects
        ens = ...
        load(strcat('D:\Projection_ML\Trained_ensemble\',Model_name{ii},'\ens_',num2str(i)));
        % Saving tunned hyperparameters
        MinObjective(i,1) = ens.ens.HyperparameterOptimizationResults.MinObjective;
        Method{i,1} = ...
        able2array(ens.ens.HyperparameterOptimizationResults.XAtMinObjective(1,1));
        Num_learning_cycles(i,1) = ...
        able2array(ens.ens.HyperparameterOptimizationResults.XAtMinObjective(1,2));
        Learn_rate(i,1) = ...
        able2array(ens.ens.HyperparameterOptimizationResults.XAtMinObjective(1,3));
        Min_leaf_size(i,1) =  ...
        able2array(ens.ens.HyperparameterOptimizationResults.XAtMinObjective(1,4));
        Max_num_splits(i,1) = ...
```

```
        able2array(ens.ens.HyperparameterOptimizationResults.XAtMinObjective(1,5));
        Num_variables_to_sample(i,1) = ...
        able2array(ens.ens.HyperparameterOptimizationResults.XAtMinObjective(1,6));
        % Validation of the trained ensemble using 10-fold cross-validation
        cvens = crossval(ens.ens,'Kfold',10);
        yfit = kfoldPredict(cvens);
        mse(i,1) = mean((ytrue - yfit).^2);
        mae(i,1) = mean(abs(yfit - ytrue));
        NSE(i,1) = 1 - sum((ytrue - yfit).^2)/sum((ytrue - mean(ytrue)).^2);
        % Coefficent of determination
        R2(i,1) = (sum((ytrue-mean(ytrue)).*(yfit-mean(yfit)))...
            /(sqrt(sum((ytrue-mean(ytrue)).^2))*sqrt(sum((yfit-mean(yfit)).^2))))^2;
    end
    rmse = sqrt(mse);% Root mean square error


    % Exporting the output into a table
    Statistics = [Num_learning_cycles Learn_rate Min_leaf_size Max_num_splits ...
    Num_variables_to_sample...
        mse rmse mae NSE R_2 MinObjective];
    ststistics_bootci = [mse rmse mae NSE R2 MinObjective];
    Statistics_table = array2table(Statistics,'VariableNames',{'Num_learning_cycles'
'Learn_rate'  'Min_leaf_size'...
    'Max_num_splits' 'Num_variables_to_sample'...
    'mse' 'rmse' 'mae' 'NSE' 'R2' 'MinObjective'});
    % Saving the table on disk
    Statistics_table.Method = Method;
    writetable(Statistics_table,strcat('D:\Projection_ML\Trained_ensemble\',Model_name{ii},...
    '\Bootstrapping_input.txt'));

    % % Bootstrapping
    % Computing the 95% confidence intervals of the mean for the statistics calculated
    % in the  above loop
    % using 1,000 bootstrap iterations. “bootci” creates each bootstrap sample
    % by sampling with replacement from the rows of the data arguments and
    % computes the confidence interval by bias corrected and accelerated percentile method.

    opt = statset('UseParallel',false);
    ci = bootci(1000,{@nanmean,ststistics_bootci},'type','bca','Options',opt);
    ci = array2table(ci,'VariableNames',{'mse' 'rmse' 'mae' 'NSE' 'R_2' 'MinObjective'});
    % Exporting the output into a table
    writetable(ci,strcat('D:\Projection_ML\Trained_ensemble\',Model_name{ii},'\Reg_CI.txt'));
end
```

Similar to the procedure used for input training profiles data, we extracted the values of pure spatial and spatio-temporal predictors to the locations of the drylands that we needed to make predictions for. The best fitted models were chosen from the 30 models trained for each of the 16 input datasets and used for prediction of the soil salinity (EC_e) at different depths. Different predictions were then averaged using trapezoidal rule to the depth of one meter from the surface and saved in .text tables for further analysis and calculation of the soils with salinity of $EC_e \geq 2$ dS m^{-1}. For each location, x- y- coordinates and 197 prediction from 1904 to 2100 were made. The following script shows how we merged the tables of purely spatial and spatio-temporal predictors and made predictions for soil salinity:

```
clc;
clear;

% This code merges the pure spatial and spatio-temporal predictors'
% values and use them to predict the soil salinity (ECe) at new locations

% Importing the purely spatial predictors table
Soil_pro =
readtable('D:\Projection_ML\new_data\Base_points\Spatial_preditors.txt','FileType','text','Del
imiter','comma');
Soil_pro = standardizeMissing(Soil_pro,-9999);
Soil_pro = fillmissing(Soil_pro,'nearest');
Model_name = {'Model_name_1'...
    'Model_name_2'...
    'Model_name_n'};
```

```
for ii = 1:length(Model_name) % Repeating the process for all 16 models
    % Finding the trained ensemble with the lowest RMSE
    Table = readtable(strcat('D:\Projection_ML\Trained_ensemble\', ...
    Model_name{ii},'\Bootstrapping_input.txt'),'FileType', ...
   'text','Delimiter',',','PreserveVariableNames',true);
    Table.Num = (1:30)';
    Table = sortrows(Table,'rmse','ascend');

    % Loading the best model
    Regression =  load(strcat('D:\Projection_ML\Trained_ensemble\', ...
    Model_name{ii},'\ens_',num2str(table2array(Table(1,13)))));

    % Importing the tables of spatio-temporal predictors
    dryss_table = readtable(strcat('D:\Projection_ML\new_data\',Model_name{ii},...
    '\dryss_',Model_name{ii},'.txt'),'FileType','text','Delimiter','comma');
    wetss_table = readtable(strcat('D:\Projection_ML\new_data\',Model_name{ii},...
    '\wetss_',Model_name{ii},'.txt'),'FileType','text','Delimiter','comma');
    evspsbl_table = readtable(strcat('D:\Projection_ML\new_data\',Model_name{ii},...
    '\evspsbl_',Model_name{ii},'.txt'),'FileType','text','Delimiter','comma');
    pr_mean_table = readtable(strcat('D:\Projection_ML\new_data\',Model_name{ii},...
    '\pr_mean_',Model_name{ii},'.txt'),'FileType','text','Delimiter','comma');
    pr_fre_table = readtable(strcat('D:\Projection_ML\new_data\',Model_name{ii},...
    '\pr_fre_',Model_name{ii},'.txt'),'FileType','text','Delimiter','comma');

    % Merging the tables of purely spatial and spatio-temporal predictors
    T_dryss =
join(Soil_pro,dryss_table,'LeftKeys','pointid','RightKeys','Base_points_drylands');
    T_wetss =
join(Soil_pro,wetss_table,'LeftKeys','pointid','RightKeys','Base_points_drylands');
    T_evspsbl = ...
    join(Soil_pro,evspsbl_table,'LeftKeys','pointid','RightKeys','Base_points_drylands');
    T_pr_mean = ...
    join(Soil_pro,pr_mean_table,'LeftKeys','pointid','RightKeys','Base_points_drylands');
    T_pr_fre = ...
    join(Soil_pro,pr_fre_table,'LeftKeys','pointid','RightKeys','Base_points_drylands');



    WRB = table2array(Soil_pro(:,12)); % Categorizing the categorical variables in the
    % training  set
    upper_dept = repmat([0 10 30 60 100],length(WRB),1);
    Clay = table2array(Soil_pro(:,15));
    Elevation = table2array(Soil_pro(:,14));
    Slope = table2array(Soil_pro(:,13));
    Root_depth = table2array(Soil_pro(:,5));
    Wilt_point = table2array(Soil_pro(:,8));
    Field_capa = table2array(Soil_pro(:,7));

    % Attributing spatio-temporal data to location of the points based on
    % the year of sampling the profile
    dryss = fillmissing(table2array(T_dryss(:,19:219)),'nearest');
    dryss = movmean(dryss,[4 0],2,'omitnan','Endpoints','discard');
    wetss = fillmissing(table2array(T_wetss(:,19:219)),'nearest');
    wetss = movmean(wetss,[4 0],2,'omitnan','Endpoints','discard');
    evspsbl = fillmissing(table2array(T_evspsbl(:,19:219)),'nearest');
    evspsbl = movmean(evspsbl,[4 0],2,'omitnan','Endpoints','discard');
    pr_mean = fillmissing(table2array(T_pr_mean(:,19:219)),'nearest');
    pr_mean = movmean(pr_mean,[4 0],2,'omitnan','Endpoints','discard');
    pr_fre = fillmissing(table2array(T_pr_fre(:,19:219)),'nearest');
    pr_fre = movmean(pr_fre,[4 0],2,'omitnan','Endpoints','discard');

    % Predicting the soil salinity at the five depths: 0, 10, 30, 60, and 100 cm
    % and averaging the output by the trapezoidal rule
    Salinity = zeros(length(WRB),size(dryss,2));
    Y = zeros(length(WRB),5);
    for i = 1:size(dryss,2)
        for j = 1:5
            X = [upper_dept(:,j) upper_dept(:,j) WRB Clay Elevation...
                Slope Field_capa Wilt_point Root_depth...
                dryss(:,i) wetss(:,i) evspsbl(:,i) pr_fre(:,i)...
                pr_mean(:,i)];
            X = array2table(X,'VariableNames',{'upper_dept' 'lower_dept'...
                'WRB' 'Clay' 'Elevation' 'Slope' 'Field_capa'...
                'Wilt_point' 'Root_depth' 'dryss' 'wetss' 'evspsbl' 'pr_fre' 'pr_mean'});
            X.WRB = categorical(X.WRB);
            Y(:,j) = predict(Regression.ens,X);
```

```
        end
        Salinity(:,i) = trapz([0 10 30 60 100],Y,2)/100;
    end

    % Replacing the missing values with flag of the missing values (-9999)
    Salinity = fillmissing(Salinity,'constant',-9999);

    % Preparing a tables for saving the results in
    Years = cell(1,201); count = 1904;
    for i = 5:201
        Years{i} = num2str(count);
        count = count + 1;
    end

    % Saving the table of results on disk
    Years{1} = 'pointid'; Years{2} = 'grid_code'; Years{3} = 'X'; Years{4} = 'Y';
    grid = [Soil_pro.pointid Soil_pro.grid_code Soil_pro.POINT_X Soil_pro.POINT_Y Salinity];
    T_result = array2table(grid,'VariableNames',Years);
 writetable(T_result,strcat('D:\Projection_ML\Results\',Model_name{ii},'_future{ii}','.txt'));
end
```

The following code was used for calculation of the cell area in WGS 1984 spatial coordinates system.

```
Calculating m^2 area of a WGS 1984 square pixel
% Adapted from: https://gis.stackexchange.com/a/127327/2397
% Parameters:
% cell_size (float): Pixel size in the Geographic coordinates (WGS 1984)which is 0.5 here
% Returns: Area of square pixel of side length cell_size in m^2

f_up = deg2rad(T.Y + cell_size/2);
f_down = deg2rad(T.Y - cell_size/2);
zm_up = (1 - e*sin(f_up));
zp_up = (1 + e*sin(f_up));
area_up = pi * b^2 * (log(zp_up./zm_up)/(2*e) + sin(f_up)./(zp_up.*zm_up));
zm_down = (1 - e*sin(f_down));
zp_down = (1 + e*sin(f_down));
area_down = pi * b^2 * (log(zp_down./zm_down)/(2*e) + ...
sin(f_down)./(zp_down.*zm_down));
cell_area = cell_size/360.*(area_up - area_down);
```
